# The Influence of Cell Source and Donor Age on the Tenogenic Potential and Chemokine Secretion of Human Mesenchymal Stromal Cells

**DOI:** 10.1155/2019/1613701

**Published:** 2019-05-07

**Authors:** Weronika Zarychta-Wiśniewska, Anna Burdzińska, Katarzyna Zielniok, Marta Koblowska, Kamila Gala, Piotr Pędzisz, Roksana Iwanicka- Nowicka, Anna Fogtman, Aleksandra Aksamit, Agnieszka Kulesza, Aleksandra Zołocińska, Leszek Pączek

**Affiliations:** ^1^Department of Immunology, Transplantology and Internal Medicine, Medical University of Warsaw, 02-006 Warsaw, Poland; ^2^Laboratory of Systems Biology, Faculty of Biology, University of Warsaw, 02-096 Warsaw, Poland; ^3^Laboratory of Microarray Analysis, Institute of Biochemistry and Biophysics, Polish Academy of Sciences, 02-106 Warsaw, Poland; ^4^Department of Orthopedics and Traumatology, Medical University of Warsaw, 02-005 Warsaw, Poland; ^5^Department of Regenerative Medicine, Maria Sklodowska-Curie Memorial Cancer Center, 02-781 Warsaw, Poland; ^6^Department of Bioinformatics, Institute of Biochemistry and Biophysics, Polish Academy of Sciences, 02-106 Warsaw, Poland

## Abstract

**Background:**

Cellular therapy is proposed for tendinopathy treatment. Bone marrow- (BM-MSC) and adipose tissue- (ASC) derived mesenchymal stromal cells are candidate populations for such a therapy. The first aim of the study was to compare human BM-MSCs and ASCs for their basal expression of factors associated with tenogenesis as well as chemotaxis. The additional aim was to evaluate if the donor age influences these features.

**Methods:**

Cells were isolated from 24 human donors, 8 for each group: hASC, hBM-MSC Y (age ≤ 45), and hBM-MSC A (age > 45). The microarray analysis was performed on RNA isolated from hASC and hBM-MSC A cells. Based on microarray results, 8 factors were chosen for further evaluation. Two genes were additionally included in the analysis: *SCLERAXIS* and *PPARγ.* All these 10 factors were tested for gene expression by the qRT-PCR method, and all except of RUNX2 were additionally evaluated for protein expression or secretion.

**Results:**

Microarray analysis showed over 1,400 genes with a significantly different expression between hASC and hBM-MSC groups. Eight of these genes were selected for further analysis: *CXCL6*, *CXCL12*, *CXCL16*, *TGF-β2*, *SMAD3*, *COLLAGEN 14A1*, *MOHAWK*, and *RUNX2.* In the subsequent qRT-PCR analysis, hBM-MSCs showed a significantly higher expression than did hASCs in following genes: *CXCL12*, *CXCL16*, *TGF-β2*, *SMAD3*, *COLLAGEN 14A1*, and *SCLERAXIS* (*p* < 0.05, regardless of BM donor age). In the case of *CXCL12*, the difference between hASC and hBM-MSC was significant only for younger BM donors, whereas for *COLLAGEN 14A1*—only for elder BM donors. *PPARγ* displayed a higher expression in hASCs compared to hBM-MSCs. In regard to *CXCL6*, *MOHAWK*, and *RUNX2* gene expression, no statistically significant differences between groups were observed.

**Conclusions:**

In the context of cell-based therapy for tendinopathies, bone marrow appears to be a more attractive source of MSCs than does adipose tissue. The age of cell donors seems to be less important than cell source, although cells from elder donors show slightly higher basal tenogenic potential than do cells from younger donors.

## 1. Introduction

Cell therapy is currently considered as an alternative or supportive treatment in cases of tendinopathies. It is believed that some cell types administrated into the region of injury can either directly differentiate into tenocytes or stimulate local endogenous reparative mechanisms. There are several candidate populations for such a procedure. The most important are tendon-derived cells, bone marrow-derived mesenchymal stromal (stem) cells (BM-MSCs), and adipose-derived mesenchymal stromal (stem) cells (AD-MSCs or ASCs) [1]. Tendon-derived cells possess the highest tenogenic potential among these populations [2], but human tendon tissue availability for the isolation procedure is very limited. In contrast, both BM-MSCs and ASCs can be relatively easily isolated for autologous transplantation, and additionally they are suitable for allogeneic transfers [3, 4]. Several preclinical studies suggest that injection of MSCs into injured tendon improves its healing [1]. First data from clinical trials suggest that the allogeneic MSC transplantation into affected tendon is a safe procedure and can have beneficial clinical effects [5]. There are at least two proposed mechanisms of action in which MSCs can act in tendinopathies. One concept says that MSCs can support tendon regeneration *via* direct differentiation. Indeed, it was shown that both BM-MSCs and ASCs can enter the tenogenic pathway in certain conditions *in vitro* [6, 7]. The conception of direct differentiation was recently supported by a study, in which human AD-MSCs were transplanted into rat injured tendon. Grafted cells survived for at least 4 weeks and produced tendon-associated proteins and proteoglycans which suggests tenogenic differentiation [8]. Although BM-MSCs and ASCs both belong to the MSC family, there are certain differences between these two cell populations [9, 10]. It was shown on rat cells that bone marrow-derived MSCs possess higher tenogenic potential than do adipose-derived MSCs. A similar comparison on human cells has not been previously published. Therefore, the primary aim of this study was to compare human BM-MSCs and ASCs in terms of basal tenogenic activity to provide potential clues in cell-based therapy of tendinopathies.

The second postulated mechanism of MSCs' action after transplantation is based on paracrine activity [11]. This activity is mediated by secretion of cytokines, growth factors, and chemokines [9]. It is believed that locally administrated MSCs can enhance recruitment of endogenous progenitors and in this way improve regeneration of the injured site. Also, recruitment of macrophages can be beneficial as it is known that M2 macrophages are crucial for tissue repair [12]. Additionally, MSCs were shown to drive the differentiation of macrophages into this beneficial phenotype [13]. It was previously demonstrated that the secretion of growth factors, cytokines, and metalloproteinases can differ depending on the MSCs' source [14, 15]. However, the impact of MSCs' origin on chemokine production is less examined. Therefore, we aimed to analyze chemotactic activity in MSCs from different sources.

Another unresolved issue in the MSC field concerns the impact of donor age on the cell properties. MSCs can be transplanted in an either autologous or allogeneic manner [16]. Although there is increasing amount of data that allogeneic MSC transfer is safe [3, 17], autologous therapy is still considered to be the most secure type of transplantation. However, in elder patients, the question about the impact of aging on cell features arises [18]. Therefore, the additional aim of the present study was to evaluate the influence of donor age on tenogenic marker expression and chemokine secretion.

## 2. Materials and Methods

### 2.1. Cell Isolation

#### 2.1.1. Human Bone Marrow Mesenchymal Stromal Cells (hBM-MSCs)

Bone marrow samples were obtained from 16 patients after receiving their written consent. The mean age of all BM donors was 51 years. The procedure was approved by the Local Bioethics Committee (approval number: KB/130/2013). The bone marrow was collected during standard orthopedic surgeries which required opening of the bone marrow cavity. The protocol of bone marrow MSC isolation was previously described in detail by our group [19]. Cells were cultured in standard growth medium (GM) composed of Dulbecco's modified Eagle's medium with low glucose (DMEM-LG, Sigma-Aldrich) supplemented with 15% fetal bovine serum (FBS, LONZA) and 1.5% (*v*/*v*) antibiotic–antimycotic solution (penicillin-streptomycin-amphotericin B; Invitrogen) on BD Primaria™ culture dishes (Becton Dickinson). After 2 days, GM was replenished, and after another 2 days, nonadherent cells were removed and first fibroblast-like adherent cells could be observed.

#### 2.1.2. Human Adipose Stem Cells (hASCs)

ASCs derived from 8 healthy donors were kindly provided by Prof. Pojda Z., Department of Regenerative Medicine, Maria Sklodowska-Curie Memorial Cancer Center, and Institute of Oncology. Adipose tissue was collected from healthy donors by liposuction. The samples were used in research after receiving patients' informed consent. The protocol of human adipose tissue stem cell isolation as well as identification was previously described in detail by our group [6].

During the culture of both cell types—hBM-MSCs and hASCs—the growth medium was changed every 2-3 days, rinsing previously with PBS. The passage was made after reaching subconfluence. After passage 3, cells were identified by flow cytometry and multilineage differentiation capacity.

### 2.2. Cell Identification

#### 2.2.1. *In Vitro* Osteogenic Differentiation

Cells were cultured in hMSC Osteogenic Differentiation BulletKit™ Medium (Lonza) for 3 weeks. Osteogenic differentiation was characterized by identification of mineral depositions in the extracellular matrix by staining with Alizarin Red (Sigma-Aldrich) and visualization by standard light microscopy. Additionally, the activity of alkaline phosphatase was evaluated using the semiquantitative colorimetric method. The Alkaline Phosphatase Liquid Substrate assay (Sigma-Aldrich, P7998) was used. At the end of experiment, differentiated and control cells were treated with 0.2% Triton X-100 in PBS for 20 min. The lysates, after centrifugation, were combined with reaction substrate, incubated for up to 30 min in RT, and read at 405 nm according to the manufacturer's instruction.

#### 2.2.2. *In Vitro* Adipogenic Differentiation

Cells were cultured in hMSC Adipogenic Differentiation BulletKit™ Medium (Lonza) for 3 weeks. Adipogenic differentiation was assessed using Oil Red O (Sigma-Aldrich) stain as an indicator of intracellular lipid accumulation and visualization by standard light microscopy. Additionally, on representative cell populations (two hASC populations and four BM-MSC populations, two from younger donors and two from elder donors), the effect of adipogenic differentiation on *PPARγ* gene expression was evaluated using the RT-PCR method (described in details below).

#### 2.2.3. *In Vitro* Chondrogenic Differentiation

To induce chondrogenic differentiation, three-dimensional pellet culture was performed. Unsuspended cell pellets were cultured for 19 days in the chondrogenic medium composed of DMEM high-glucose (LONZA) medium supplemented with 1% FBS, 1% (*v*/*v*) ITS+supplement, 0.1 *μ*M dexamethasone, 0.1 mM ascorbic acid 2-phosphate, 100 *μ*g/mL sodium pyruvate, and 10 ng/mL recombinant human transforming growth factor-*β*2 and 1% (*v*/*v*) penicillin-streptomycin solution. For histological analysis, pellets were immersed in paraffin, sectioned, and stained with Masson trichrome or toluidine blue method.

#### 2.2.4. Flow Cytometry Analysis

The surface antigen profiles of adipose and bone marrow-derived MSCs at the third passage were characterized by flow cytometry using Human MSC Analysis Kit (BD Biosciences). The presence of cell CD73, CD90, CD105, and CD44 and the absence of CD34, CD45, CD11b, CD19, and HLA-DR were assessed. The staining was performed in accordance with the manufacturer's instructions. Stained cells were analyzed using BD FACSCanto II (Becton Dickinson). For analysis, BD FACSDiva Software was utilized, using the same setup for all tested populations.

### 2.3. Study Design

The material was divided into 3 experimental groups:

hASC—cells from donors of unknown age, *n* = 8

hBM-MSC Y—cells from donors up to 45 years old (mean age 38.1; median age 38.0), *n* = 8

hBM-MSC A—cells from donors over 45 years old (mean age 64.4; median age 64.5), *n* = 8

Microarray analysis was performed on RNA derived from hASC and hBM-MSC A groups. From genes with significant difference in expression between the analyzed groups, several were selected for further analysis. At this stage, the cells from young BM-MSC donors were added to the study (hBM-MSC Y group). For all analyses, cells from the 4th to 6th passage were used. Experiments were always conducted on cells from each donor separately. The cells from different donors were not pooled in this study to enable the detection of interindividual differences. Cells were always cultured in sets which included 1 hASC population, 1 hBM-MSC Y population, and 1 hBM-MSC A population to avoid variability of the analysis conditions.

### 2.4. RNA Isolation

The total RNA isolation was performed using RNeasy Mini Kit (Qiagen) according to the manufacturer's instructions. At least 3 × 10^5^ cells were used for this procedure. RNA concentration and purity were assessed by spectrophotometer at 260 nm using NanoDrop (ND-1000 Spectrophotometer, NanoDrop Technologies Inc.).

### 2.5. Microarray Analysis

Microarray expression analysis was performed using the Affymetrix GeneAtlas system according to the manufacturer's instructions. 10 ng of total RNA that passed initial quality control screen (2100 Bioanalyzer, Agilent) was then processed using GeneChip™ WT Pico Kit, designed specifically to process small amounts of input RNA, according to the standard protocol provided by Affymetrix. Samples were hybridized to the GeneChip™ Human Gene 2.1 ST Array Strip (Affymetrix).

The microarrays were scanned with Affymetrix GeneAtlas Scanner, and the intensity signals for each of the probe set were written by Affymetrix software into the CEL files. The CEL files were imported into the Partek Genomics Suite v 6.6 software with the use of RMA (Robust Multiarray Averaging). During this step, background correction was applied based on the global distribution of the PM (perfect match) probe intensities and the affinity for each of the probes (based on their sequences) was calculated. Further, the probe intensities were quantile-normalized [20] and log2 transformed, and median polish summarization to each of the probe sets was applied. Then, the qualitative analysis was performed, e.g., principal component analysis, in order to identify outliers and artifacts on the microarray. After quality check, the 2-way analysis of variance (ANOVA) model by using Method of Moments [21] was applied to the data, which allowed creating lists of significantly and differentially expressed genes between the biological variants (with the cutoff values: *p* value <0.05; −1.5 > fold change > 1.5).

### 2.6. Real-Time qPCR Analysis

Specific TaqMan® Gene Expression Assays were purchased from Applied Biosystems: Mohawk homeobox (*MKX*) Hs00543190_m1, scleraxis (*SCX*) Hs03054634_g1, collagen type XIV alpha 1 (*COL14A1*) Hs00964045_m1, transforming growth factor beta 2 (*TGF-β2*) Hs00234244_m1, SMAD family member 3 (*SMAD3*) Hs00969210_m1, Runt-related transcription factor 2 (*RUNX2*) Hs01047973_m1, peroxisome proliferator-activated receptor gamma (*PPARG*) Hs00234592_m1, C-X-C motif chemokine ligand 6 (*CXCL6*) Hs00605742_g1, C-X-C motif chemokine ligand 16 (*CXCL16*) Hs00222859_m1, and C-X-C motif chemokine ligand 12 (*CXCL12*) Hs03676656_mH. Human *GUSB* was used for normalization. Real-time PCR was performed on ABI Prism 7500 Sequence Detection System using TaqMan® RNA-to-C_T_™ *1-Step* Kit (Applied Biosystems). Each sample was analyzed in duplicate. The relative gene expression was calculated by 2^−ΔΔC^t method. The results were presented as a fold change of gene expression in

hBM-MSC Y and hBM-MSC A, where the reference point was expression in hASCs

hBM-MSC A, where the reference point was expression in hBM-MSC Y

Statistical analysis was performed by comparison of ΔCt values using tests for independent samples (hASCs vs. hBM-MSC Y or A and hBM-MSC Y vs. hBM-MSC A).

### 2.7. Secretion Analysis

The cells were seeded on 12-well plates. After reaching subconfluence, the growth medium was changed to DMEM+3.5% BSA for 48 hours. After this time, the supernatants were collected, centrifuged (sediment potentially containing cell debris was discarded), aliquoted, and frozen in −80°C. After collecting the supernatants, the cells were detached and counted which enabled normalization of the secretion results to the number of the cells. The supernatants were used to assess the secretion of chosen factors: CXCL6, CXCL12, and CXCL16 were determined using the Bio-Plex Pro Human Chemokine Assay (Bio-Rad Laboratories Inc.) and TGF-*β*2 using ELISA kit (R&D Systems®) according to the manufacturer's instructions.

### 2.8. Western Blot (WB)

Collected cell pellets of human BM-MSCs and ASCs were lysed with RIPA buffer (Sigma-Aldrich) supplemented with cocktails of protease and phosphatase inhibitors (Sigma-Aldrich). Protein extraction was performed for 30 min at 4°C. Next, lysates were cleared for 20 min at 14000 rpm, and supernatants were collected. The total protein concentration was determined using Bio-Rad protein assay dye reagent (Bio-Rad Laboratories Inc.) according to the producer's instructions. Proteins (40 *µ*g of total protein per well) were resolved by SDS-PAGE and transferred onto the PVDF membrane (Millipore). For immunostaining, membranes were blocked with 5% nonfat dried milk in TBS (20 mM Tris-HCl, 500 mM NaCl) containing 0.1% Tween 20. The membranes were incubated with rabbit polyclonal anti-COL14A1 (Abcam, ab101464, 1 : 500), rabbit polyclonal anti-MOHAWK (LSBio, aa46-75, 1 : 1000), rabbit polyclonal anti-SMAD3 (Abcam, ab28379, 1 : 1000), anti-SCLERAXIS (Thermo Fisher, PA5-23943, 1 : 1000), and mouse monoclonal anti-*β*-ACTIN (Santa Cruz Biotechnology, sc47778, 1 : 1000) primary antibodies. Next, the blots were washed three times in TBST and incubated with appropriate secondary antibodies conjugated with IR fluorophores: IRDye 680 or IRDye 800 (LI-COR Biosciences) at 1 : 5000 dilution. Odyssey Infrared Imaging System (LI-COR Biosciences) was used to analyze the relative protein expression. Quantification of the integrated optical density (IOD) of target proteins was normalized to the IOD of *β*-actin and performed with the use of Odyssey analysis software (LI-COR Biosciences). Immunoblot assay for cells from each donor was performed in triplicate. For the purpose of publication, the color immunoblot images were converted into black and white images in the Odyssey software.

### 2.9. Migration Assay

The ability of analyzed populations to stimulate the directed migration of other cells was studied using cell culture inserts with 8 *μ*m pore size (ThinCert™ Greiner). The assay is aimed at simulating the recruitment of circulating or local endogenous mesenchymal cells. Cells from 4 different BM-MSC populations were pooled and stained with red fluorochrome DilC18(5)-DS (DID) (1,1′-dioctadecyl-3,3,3,-tetrametlylindodicarbocyanine-5,5-disulfonic acid), Ex = 650 nm, Em = 670 nm (AAT Bioquest). This pool constituted a migrating population in this experiment and was the same for all probes. Approximately 1 × 10^4^ of these labeled cells were seeded on each insert. In the basal compartment, there was either no cells (unstimulated migration, control) or unlabeled hASCs, hBM-MSCs Y, or hBM-MSCs A seeded in concentration 2 × 10^4^/well on a 24-well bottom plate. Each population was seeded in duplicates. Cells were incubated at 37°C and 5% CO_2_ for the next 48 h to allow cell migration from the inserts to the basal compartment. Afterwards, inserts were removed, cells on the bottom of the wells were fixed with 4% paraformaldehyde (10 min, RT), and the cell nuclei were visualized with DAPI staining (20 ng/mL of DAPI solution for 4 minutes, RT). Additionally, migrating cells were trypsinized from the bottom side of inserts, they were allowed to attach overnight, and they also fixed and stained with DAPI. The cells were visualized and with imaging reader Cytation™ 1 (BioTek) and counted with Gene5 3.04 software (Figure S1). The number of migrating/stimulating cells was calculated from 25 different fields of view from each well in each sample. The migrating cells constituted the sum of those that fell from the insert and attached to the well bottom during experiment duration (DIDf) and those that were detached from the underside of an insert (DIDins) and attached after the experiment was finished. Fields of view had the same locations in all wells and were chosen arbitrarily before analysis. As the number of stimulating cells differed at the end of experiment between different cell populations, for the final analysis we have chosen those populations in which the number of stimulating cells (after automatic calculation) was the most similar (one population from each cell type). Two different analyses were performed. First, the number of migrating cells for each type of stimulus was compared to the control (unstimulated migration). The sum of DIDf and DIDins was taken for this analysis. Second, stimulating cell types were compared between each other. We called this parameter “stimulation potential.” For this analysis, the ratio of migrating cell (with red fluorescence, Figure S1A') number to the stimulating cell number (unlabeled, Figure S1A”) was calculated in accordance to the equation below.

(1) $$\begin{array}{c} \text{Stimulation potential}=\frac{\left(\text{DIDf}+\text{DIDins}\right)}{\left(\text{DAPI}-\text{DIDf}\right)}*100, \end{array}$$

where

DIDf—the number of migrating cells (stained with DID) which fell from the insert

DIDins—the number of migrating cells (stained with DID) which were detached from the underside of an insert

DAPI—the number of cell nuclei (stained with DAPI). Nuclei of stimulating cells and DIDf cells were counted.

### 2.10. Statistical Analysis

For data analysis, STATISTICA software (StatSoft® Polska) was used. Data are presented as means ± SD or means ± SEM. Differences between groups were analyzed by Student *t*-test or Mann–Whitney *U* test depending on data distribution in analyzed groups. Verification of the hypothesis of characteristic normal distribution in the analyzed populations was performed using the Shapiro-Wilk test for each analyzed group of data. The hypothesis of variance homogeneity was verified using Levene's test. *p* < 0.05 was considered as statistically significant.

## 3. Results

### 3.1. Isolation and Identification

**Figure 1:**
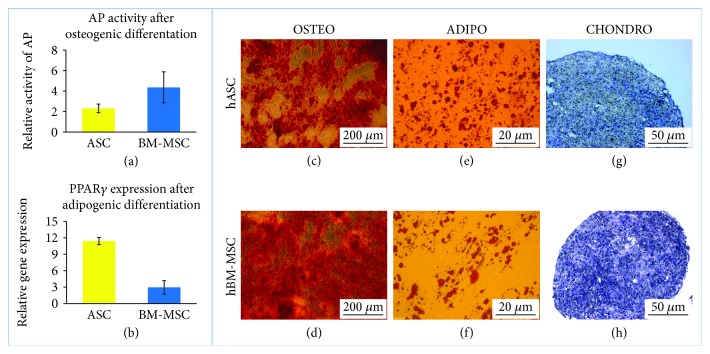
Human MSC differentiation potential. Osteogenic differentiation: (a) change in alkaline phosphatase activity in cells subjected to osteogenic differentiation in relation to the nondifferentiated control for hASC populations, for hBM-MSCs 3 populations of hBM-MSC Y and 3 pop hBM-MSC A; (c, d) light microscopy, Alizarin Red staining (calcium deposits are red) of hMSCs cultured in standard osteogenic medium; adipogenic differentiation: (b) *PPARγ* gene expression determined by the RT-PCR method and calculated using the 2^−ΔΔCt^ method. Results presented as fold change in relation to the expression in nondifferentiated control; (e, f) light microscopy, Oil Red O staining (lipid droplets are red) of hMSCs cultured in adipogenic medium; (g, h) chondrogenic differentiation, toluidine blue staining of chondropellet, collagen II fibers are blue; representative pictures of adipose tissue and bone marrow-derived cells are presented. Scale bars: 200 *μ*m (c, d); 20 *μ*m (e, f); 50 *μ*m (g, h). *PPARγ*: *PEROXISOME PROLIFERATOR ACTIVATED RECEPTOR GAMMA*; AP: alkaline phosphatase. Data presented as mean ± SEM.

Cells have been successfully isolated from 24 donors (8 donors for each group). The cells exhibited adherent properties and the ability to form colonies and to differentiate on the adipo-, osteo-, and chondrogenic pathways (Figure 1). Cells isolated from the bone marrow appeared to be more prone to differentiate into osteogenic lineage than did cells isolated from adipose tissue (Figure 1(a)). An opposite trend was observed in regard to the adipogenic differentiation (Figure 1(b)). Flow cytometry analysis confirmed the expression of CD90 (median of 100%, 98%, and 98.9% of positive cells in hASCs, hBM-MSC Y, and hBM-MSC A, respectively), CD44 (median of 99.5%, 99.3%, and 99% of positive cells in hASCs, BM-MSC Y, and BM-MSC A, respectively), CD105 (median of 100%, 98.9%, and 98.7% of positive cells in hASCs, BM-MSC Y, and BM-MSC A, respectively), and CD73 (median of 99.5%, 99.7%, and 99.6% of positive cells in hASCs, BM-MSC Y, and BM-MSC A, respectively).

### 3.2. Microarray Analysis

Microarray analysis revealed over 1,400 genes of significantly different expression between hASCs and hBM-MSCs A. The list of these genes is presented in Figure S2 in the Supplementary Material (available here). We were particularly interested in genes associated with tenogenesis and chemotaxis. The tenogenesis-associated genes with a significantly different expression in analyzed groups included *TGF-β2* (4.5-fold difference in expression), *COL14A1* (3.9-fold), *SMAD3* (2.9-fold), *LUMICAN* (2.3-fold), *GROWTH AND DIFFERENTIATION FACTOR 5* (2.08-fold), *MKX* (1.96-fold), *COL6* (1.96-fold), *DECORIN* (1.8-fold), *FIBROBLAST GROWTH FACTOR (FGF) RECEPTOR 1* (1.89-fold), *FGF9* (1.6-fold), *ELASTIN* (1.5-fold), and *FGF10* (1.5-fold) (ranked according to the fold change difference). All these genes except *FGF9* displayed a higher expression in BM-MSCs than in ASCs. In regards to chemotaxis—there were 7 chemokines with significantly different expression in analyzed groups: *CXCL16* (10-fold difference in expression), *CXCL6* (5-fold), *CXCL12* (3.6-fold), *CCL2* (3.5-fold), *CXCL8* (2.8-fold), *CXCL1* (2.5-fold), and *CCL4* (2-fold)—all of them displayed a higher expression in BM-MSCs than in ASCs. From the obtained database, 8 factors associated with areas mentioned above were selected for further analysis. There were 3 tenogenesis-associated genes with the highest fold change expression based on microarray: *TGF-β2*, *COL14A1*, and *SMAD3*. To this group, *MKX* was added as one of crucial tenogenic transcription factors. Next, there were 3 genes encoding chemokines with the highest fold change expression based on microarray: *CXCL16*, *CXCL6*, and *CXCL12*. To this list, we added *RUNX2* (1.9-fold difference) as we aimed to evaluate the activity of the competitive osteogenic pathway. Two genes were additionally included in the analysis: *SCX* and *PPARγ*. This allowed for testing in various directions:

1. Genes related to chemotaxis and cell recruitment (*CXCL6*, *CXCL16*, and *CXCL12*)
2. Genes related to tenogenic differentiation potential (*MKX*, *COL14A1*, *TGF-β2*, *SMAD3*, and *SCX*)
3. Transcription factors associated with the competitive osteogenic and adipogenic pathways (*RUNX2*, *PPARγ*)

### 3.3. The Effect of Cell Type on Migration and Cell-to-Cell Interaction

**Figure 2:**
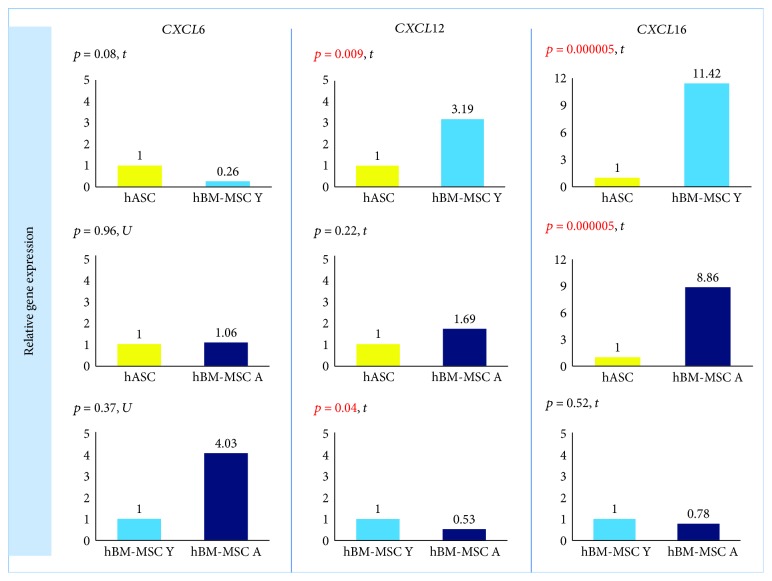
hMSCs' origin affects expression of genes related to chemotaxis. Gene expression determined by the RT-PCR method and calculated using the 2^−ΔΔCt^ method. Results presented as fold change in relation to the expression in hASCs or hBM-MSC Y whose value was taken as 1. Statistical analysis was performed by comparison of dCt values by Student's *t*-test (*t*) or Mann–Whitney *U* test (U). *p* < 0.05 was assumed to be statistically significant and highlighted in red; *CXCL6*: C-X-C *MOTIF CHEMOKINE LIGAND* 6; *CXCL12*: C-X-C *MOTIF CHEMOKINE LIGAND* 12; *CXCL16*: C-X-C *MOTIF CHEMOKINE LIGAND 16*.

**Figure 3:**
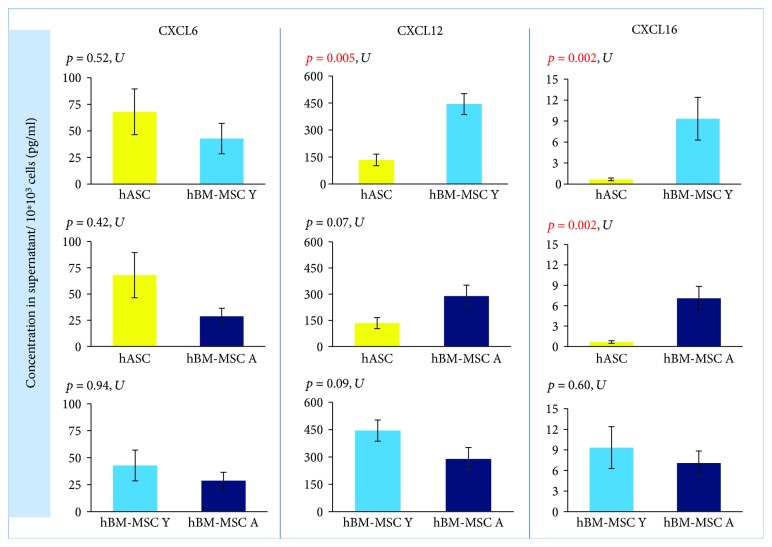
The hMSCs' origin influences secretion of chemokines. Chemokine secretion assessed using Luminex. Results shown as the concentration in the supernatant per 10^∗^10^3^ cells. Data presented as mean ± SEM. Statistical analysis was performed by Mann–Whitney *U* test (U). *p* < 0.05 was assumed to be statistically significant and highlighted in red. CXCL6: C-X-C MOTIF CHEMOKINE LIGAND 6; CXCL12: C-X-C MOTIF CHEMOKINE LIGAND 12; CXCL16: C-X-C MOTIF CHEMOKINE LIGAND 16.

In qRT-PCR analysis, cells isolated from the bone marrow showed a statistically significant higher expression of *CXCL16* (*p* = 0.000005) than did hASCs. The fold change for *CXCL16* was 11.42 (hBM-MSC Y) and 8.86 (hBM-MSC A) compared to hASC = 1 (Figure 2). Coherence of results was found regardless of the hBM-MSCs donors' age. The secretion study confirmed the results of the gene expression study —CXCL16 was significantly less secreted in hASCs than in hBM-MSCs (the age of donors was negligible) (Figure 3). In contrast, the level of *CXCL12* expression was significantly different only between hASCs and BM-MSCs from younger donors (*p* = 0.009) and not between hASC and hBM-MSC A. In addition, a significant difference was observed for *CXCL12* from hBM-MSC depending on donors' age (*p* = 0.04) (Figure 2). However, in the case of CXCL12 secretion, differences between hBM-MSC Y and hBM-MSC A groups were negligible (Figure 3). In regard to *CXCL6* gene expression, no statistically significant differences were found for all analyzed variants (marrow vs. fat, young vs. aged) in the case of gene expression analysis (Figure 2) as well as secretion (Figure 3).

### 3.4. The Origin of Cells Affects the Expression/Secretion of Factors Associated with Tenogenesis

**Figure 4:**
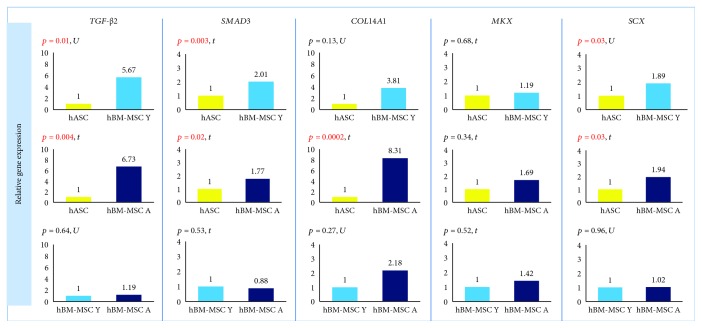
The origin of hMSCs affects the expression of factors associated with tenogenesis. Gene expression determined by the RT-PCR method and calculated using the 2^−ΔΔCt^ method. Results presented as fold change in relation to the expression in hASCs or hBM-MSC Y whose value was taken as 1. Statistical analysis was performed by comparison of dCt values by Student's *t*-test (*t*) or Mann–Whitney *U* test (U). *p* < 0.05 was assumed to be statistically significant and highlighted in red. *TGF-β2*: *TRANSFORMING GROWTH FACTOR BETA 2*; *SMAD3*: *SMAD FAMILY MEMBER 3*; *COL14A1*: *COLLAGEN TYPE XIV ALPHA 1*; *MKX*: *MOHAWK HOMEOBOX*; *SCX*: *SCLERAXIS*.

**Figure 5:**
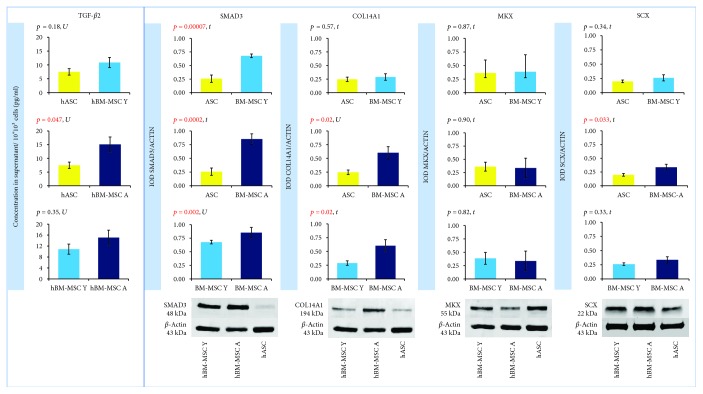
The origin of cells affects the formation of proteins associated with tenogenesis. Left panel: TGF-*β*2 secretion determined by ELISA. Results shown as the concentration in the supernatant per 10^∗^10^3^ cells. Right panel: SMAD3, COL14A1, MKX, and SCX determined by Western blot. Expression of *β*-actin was used as a loading control. The results shown as integrated optical density (IOD) normalized to IOD of corresponding *β*-actin. Data presented as mean ± SEM. Statistical analysis was performed by Student's *t*-test (*t*) or Mann–Whitney *U* test (U). *p* < 0.05 was assumed to be statistically significant and highlighted in red. TGF-*β*2: TRANSFORMING GROWTH FACTOR BETA 2; SMAD3: SMAD FAMILY MEMBER 3; COL14A1: COLLAGEN TYPE XIV ALPHA 1; MKX: MOHAWK HOMEOBOX; SCX: SCLERAXIS.

The expression of *TGF-β2* and *SMAD3* genes was significantly higher in hBM-MSCs than in hASCs regardless of BM-MSCs' donor age. The calculated fold change for *TGF-β2* was 5.67 (hBM-MSC Y with reference to hASC, *p* = 0.01) and 6.73 (hBM-MSC Y vs. hASC, *p* = 0.004). For *SMAD3*, fold change was 2.01 (hBM-MSC Y vs. hASC) and 1.77 (hBM-MSC Y vs. hASC) with the level of significance *p* = 0.003 and *p* = 0.02, respectively (Figure 4). The analysis of protein expression for SMAD3 confirmed these results. Western blot analysis showed that SMAD3 is significantly less expressed in hASCs than in hBM-MSCs Y (*p* = 0.00007) and hBM-MSC A (*p* = 0.0002), and the age of donors was irrelevant (Figure 5). The differences in TGF-*β*2 secretion were statistically significant only for the hASC vs. hBM-MSC A group (*p* = 0,047) (Figure 5). BM-MSCs showed a statistically significant higher expression of *SCX* associated with tenogenesis than hASCs (*p* = 0.03 in both Y and A groups, fold change 1.89 and 1.94, respectively), and it did not depend on the age of bone marrow donors (Figure 4). In contrast, another transcription factor important for tenogenesis—*MKX*—did not differ significantly between groups at both of the gene expression (Figure 4) and protein level (Figure 5). A higher expression of *COL14A1* has been demonstrated in hBM-MSCs A (*p* = 0.0002, fold change 8.31) than in cells derived from adipose tissue (Figure 4). In the case of *COL14A1*, despite the fold change obtained at 3.81, differences in expression between hASC and hBM-MSC Y were not statistically significant. Similarly, for *COL14A1* there were no significant differences regarding the age of the bone marrow donor. At the protein level, there were no differences between hBM-MSC Y and hASCs (Figure 5). A statistically significant increase was achieved in the hBM-MSC A group compared to hASCs (*p* = 0.02). Additionally, a significant difference in protein expression was observed for COL14A1 from hBM-MSC depending on donors' age and was higher in the hBM-MSC A group (*p* = 0.02) (Figure 5).

### 3.5. The Effect of Cell Origin on Expression of Factors Associated with Osteogenesis and Adipogenesis

**Figure 6:**
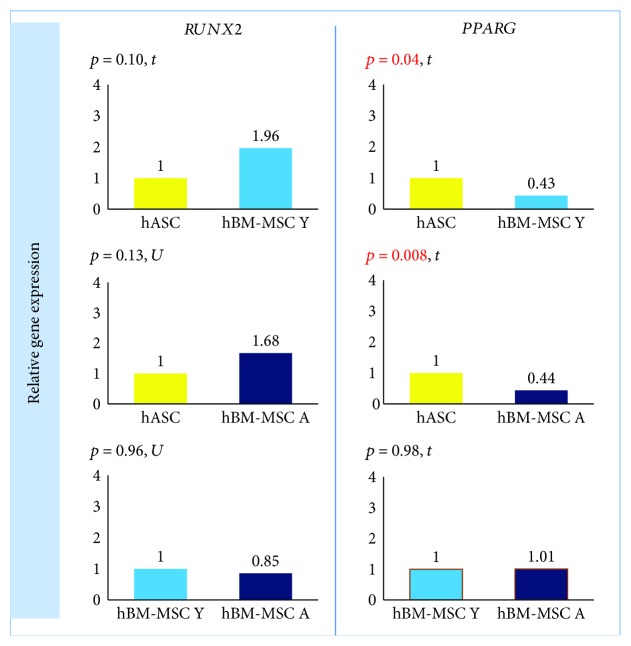
The origin of hMSCs affects the expression of transcription factors associated with the differentiation pathways. Gene expression determined by the RT-PCR method and calculated using the 2^−ΔΔCt^ method. Results presented as fold change in relation to the expression in hASCs or hBM-MSC Y whose value was taken as 1. Statistical analysis was performed by comparison of dCt values by Student's *t*-test (*t*) or Mann–Whitney *U* test (U). *p* < 0.05 was assumed to be statistically significant and highlighted in red. *RUNX2*: *RUNT-RELATED TRANSCRIPTION FACTOR 2*; *PPARG*: *PEROXISOME PROLIFERATOR ACTIVATED RECEPTOR GAMMA.*

*PPARG* expression was significantly lower in hBM-MSC Y and hBM-MSC A than in hASCs (fold change 0.43, *p* = 0.04, and fold change 0.44, *p* = 0.008, respectively) with no differences between the bone marrow groups (Figure 6). Although the mean expression of *RUNX2* was 1.96- and 1.68-fold higher in hBM-MSC (Y and A, respectively) than in hASCs, there were no significant differences in *RUNX2* expression between groups (Figure 6).

### 3.6. The Effect of Cells' Origin on Directed Migration of BM-MSCs

**Figure 7:**
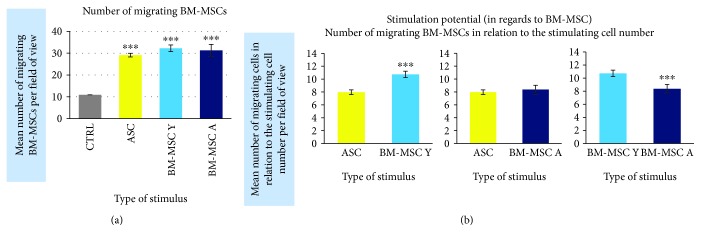
The origin of MSCs affects the chemotactic potential in relation to BM-MSC. Results based on 8 *μ*m insert migration assay. (a) The number of migrating cells stimulated by different cell types in comparison to the unstimulated control (CTRL, migration to the empty well). (b) The number of migrating cells in relation to the number of stimulating cells calculated at the end of experiment. Calculated from 50 different, arbitrary chosen fields of view for each cell type. Data presented as mean ± SEM. Statistical analysis was performed using Mann-Whitney *U* test. ^∗∗∗^*p* < 0.001.

Our functional analysis demonstrated that MSCs regardless of cell origin highly significantly stimulate the migration of BM-MSC. The mean number of migrating BM-MSCs increased in the presence of stimulating cells by 2.9-fold, 3.2-fold, and 3.1-fold (for ASC, BM-MSC Y, and BM-MSC A, respectively) in relation to unstimulated control (*p* < 0.001 for all groups, Figure 7(a)). The additional analysis took into account the differences in the number of stimulating cells calculated at the end of the experiment as presented in “Materials and Methods”. At least 1400 of migrating cells from each cell type were included to this analysis. It revealed that the stimulatory potential in regard to BM-MSCs of BM-MSC derived from young donors was by 34.7% and 28.2% higher than the potential of ASC and BM-MSC A, respectively (*p* < 0.001 in both cases). The potential of ASC and BM-MSC A did not differ significantly.

## 4. Discussion

Mesenchymal stromal cells are under testing in the treatment of wide range of disorders (https://clinicaltrials.gov). Although a spectacular number of studies have been already conducted on MSCs, there are still many issues which remain to be elucidated. Mesenchymal stromal cells isolated from different tissues were shown to share general features, which enable classifying them as MSCs. However, there is growing amount of data indicating that the source of MSCs does matter for their detailed characteristic [14, 22, 23]. Therefore, it seems to be very important to perform comparative studies on different MSC types in order to evaluate their utility for particular clinical applications. In the present study, the main objective was to compare human BM-MSCs and ASCs in terms of potential utility in cell-based therapy of tendinopathies. Of course, there is little chance to replicate the normal (prenatal) pattern of tendon formation by cell-based therapy considering that genes responsible for tenogenesis are associated with at least 400 canonical pathways [24]. The purpose of cellular transplantation in tendon injuries is rather to support natural, imperfect tendon healing and to obtain functional structures with high resistance to re-injury. Previously, the tenogenic potential of MSCs isolated from different sources was studied only on animal cells [6, 7, 25]. The present study was planned and conducted to evaluate the human MSCs in the three-step protocol including the microarray analysis, the evaluation of gene expression level using RT-PCR, and finally the assessment of protein product expression or secretion (depending on a certain protein function). This kind of study design provides high-quality data. Moreover, we have used cells from 24 independent donors (*n* = 8 per group), which additionally increases the reliability of obtained results. In our comparative analysis of hASCs and hBM-MSCs, over 1400 genes showed a significantly different expression. It accounted for 2.6% of all analyzed transcripts. It is a meaningful number of genes, especially if taking into account the interindividual diversity of MSCs. From this large group of genes, several have been selected for further analysis. We were particularly interested in genes which are associated with tenogenesis and chemotaxis.

The crucial component of a tendon tissue is collagen type I. Other collagens associated with the tenogenesis are collagens type III, V, VI, XII, and XIV [26]. Our comparative microarray analysis demonstrated that only one of these collagens displayed a significantly different expression in hBM-MSCs and hASCs. It was *COL14A1*, which showed a 3.9-fold higher expression in hBM-MSCs than in hASCs. In another work, *Col14* was also shown to be highly upregulated in equine BM-MSCs cultured in tenogenic conditions [27]. The authors indicated that increased *Col14* expression in BM-MSC was associated with the Wnt/*β*-catenin pathway. Activation of this type of signaling increased also the expression of other genes associated with tenogenesis: *tenomodulin*, *decorin*, and *fibromodulin.* The formation of collagen type I and type XIV is dependent on the expression of *Scleraxis* [28]. Our results demonstrate that BM-MSCs displayed a significantly higher basal expression of *SCLERAXIS*, which was confirmed on a protein level for the BM-MSC A group. This transcription factor was shown to be critically involved in embryonic tendon development and plays a pivotal role in the fate determination of MSCs towards tenocyte differentiation [29]. Additionally, deficiency of *Scleraxis* causes increased secretion of *Sox 9* and promotion of the chondrogenesis path [28]. Therefore, our results suggest that human bone marrow-derived MSCs possess higher tenogenic potential than ASCs. Similar conclusions were previously driven from studies on mouse, rat, or equine MSCs [7, 30, 31]. In horses, it was also shown that BM-MSC transplantation into experimentally injured tendon provided a better outcome than analogous transplantation of ASC [32]. It is therefore, possible, that bone marrow can be a more desired source of MSC for potential treatment of tendon injuries also in human patients. Dai et al. [7] demonstrated that rat BM-MSCs show a significantly higher basal expression of *Scleraxis* and *Collagen I* in comparison to adipose tissue MSCs and that BM-MSCs displayed a significantly more pronounced response to tenogenic BMP-12 treatment than ASCs did. Moreover, BMP-12-treated hASCs showed lower *Scleraxis* expression than the untreated BM-MSCs did [7]. In an earlier study of our group [6], the induction of human ASCs by 100 ng/mL of BMP-12 for 7 days resulted in 2.05 expression fold change of *SCLERAXIS.* In the present study, we show that the baseline expression of *SCLERAXIS* is almost 2-fold higher in hBM-MSCs than in hASCs. Those results taken together suggest that activity of the tenogenic pathway in hASCs after 7 days of treatment can be comparable to the one in nontreated hBM-MSCs. Our group has previously shown that induction of tenogenesis by using pleiotropic factors from the TGF-*β* family may influence other MSCs' features relevant to the fate of cells after transplantation. The use of BMP-12 caused the impairment of immunomodulatory properties of treated MSCs and affected the secretion of IL-6 and VEGF by these cells [6]. Therefore, an interesting alternative to the use of *in vitro* predifferentiated cells for transplantation would be to use cells with initially more favorable features in a given application. An even more advanced approach could be drawn based on results presented by Hou et al. [33]. They demonstrated that within BM-MSCs, there is a subpopulation that physiologically expresses a high level of *tenomodulin* (*Tnmd*). The authors postulated that selecting the natural tenogenic subpopulation from BM-MSCs could be a better solution than using tenogenic *in vitro* pretreatment before transplantation. Indeed, these cells were more susceptible to tenogenic induction and displayed an increased expression of tenogenic factors—BMP-12 and BMP-13 than BM-MSCs with normal *Tnmd* expression. Moreover, cells with a high expression of *Tnmd* possessed an upregulated TGF-*β* signaling pathway. TGF-*β*/SMAD2/3 and ERK MAPK signaling pathways seem to be crucial for the formation of tendons [24]. Studies in mice, rats, and chicks showed that TGF-*β*1, 2, and 3 have a positive and significant effect on the tenogenic differentiation in tendon stem/progenitor cells, ADSCs, and amniotic fluid stem cells [24, 34, 35]. Supplementation of TGF-*β* increased the *Scleraxis*, *Collagen I*, and *Mohawk* expression in these cells. In addition, TGF-*β* promoted the tenogenesis pathway, decreasing *Sox9* expression, which is a cartilage formation marker [24]. This is important because with the intersecting signaling pathways and many coacting factors involved in tendon formation, there is a risk of activating the undesired path in *in vitro* treated cells. In our study, the level of TGF-*β*2 expression was significantly higher in bone marrow-derived cells than in adipose tissue-derived MSCs at the microarray analysis phase (fold change 4.55, *p* < 0.00008). This result was confirmed in evaluation of gene expression by the qRT-PCR method. Moreover, BM-MSCs also showed a significantly higher expression of SMAD3 in both gene and protein expression analyses. Our data indicate that the TGF-*β* pathway can be generally more active in BM-derived cells than in adipose tissue-derived MSCs which can be associated with enhanced tenogenic potential.

In this study, we were also interested if the expression of chemokines varies in MSCs from different sources. There is a growing amount of evidence that paracrine effect has significant importance in therapeutic MSCs' mechanism of action. Among a wide panel of factors secreted by MSCs, the chemotactic cytokines (chemokines) seem to be a relevant, but relatively little researched part. The process of inflammation, in which chemokines drive the recruitment and migration of cells, is a necessary step in tissue regeneration/healing. On the other hand, excessive or prolonged inflammatory reaction can lead to pathologic consequences. The influence of immune cells (especially macrophages) on the process of regeneration was intensively studied and confirmed in skeletal muscle tissue [36]. Tendon healing is also modulated by the immune system [37], but the naturally occurring process is slow and ends up with impaired tendon strength. It is assumed that modification of inflammatory response by MSC-based therapy can improve tendon healing. Our comparative analysis of hASC and hBM-MSC transcriptomes using Affymetrix microarray revealed 7 genes encoding chemokines with a significantly different expression in these two cell types. The group of genes included *CXCL16*, *CXCL6*, *CXCL12*, *CCL2*, *CXCL8*, *CXCL1*, and *CCL4*. In all cases, the expression was higher in hBM-MSCs suggesting that BM-MSCs are generally more active in secreting chemokines than ASCs. Taking into account the physiological role of BM-derived stromal cells, it is not a surprising result. Three chemokines with the highest fold change in gene expression difference were selected for further analysis. In the case of CXCL16, the results of microarray were unequivocally confirmed in subsequent tests. Bone marrow-derived MSCs displayed a significantly higher expression of *CXCL16* than did hASCs (*p* < 0.00001 regardless of BM donor age) which was further confirmed by evaluation of CXCL16 in cell culture supernatants. CXCL16 is the only ligand for the CXCR6 receptor. The CXCR6/CXCL16 axis has a rather bad press as its increased activity was shown to be associated with invasion of some cancers [38] and development of cardiometabolic disorders [39]. However, on the other hand, CXCL16 was demonstrated to be a critical mediator of muscle regeneration, which suppresses the development of fibrosis [40]. In mice lacking CXCL16, the infiltration of macrophages to the injured muscle was impaired and macrophages were shown to be a necessary element in skeletal muscle regeneration. It is not clear how an increased level of CXCL16 would affect the tendon healing and it clearly requires further investigation. It could be assumed that it could enhance monocyte/macrophage recruitment to the site of injury. It is postulated that MSCs drive differentiation of macrophages into the M2 phenotype [13], which is generally known to be crucial in inflammation resolving, tissue remodeling, and regeneration. The role of macrophages in tendon repairing is not as well recognized as in muscle tissue, but most of published data indicate that this cell population is beneficial for the tendon healing process [41, 42]. Another chemokine which displayed a higher expression in BM-MSCs than in ASCs in our microarray analysis is CXCL12 (SDF-1). This result was confirmed by the RT-PCR method, but only for hBM-MSCs derived from younger donors (3.2-fold difference in expression between hBM-MSC-Y and hASC, *p* < 0.01). Similarly, the secretion of CXCL12 was significantly higher in young (but not aged) donor-derived hBM-MSCs than in hASCs. CXCL12 is constitutively secreted in the bone marrow to enable retainment of stem and progenitor cells in the marrow, whereas adipose tissue is not a significant source of this chemokine in physiological conditions. Our study for the first time demonstrated that isolated and cultured human BM-MSCs express and secrete significantly more CXCL12 than do ASCs. We additionally demonstrated in a functional test that although all analyzed MSC types chemoattracted BM-MSCs, BM-MSC Y provided a stronger signal than did ASC or BM-MSC A. It is probable that this effect was due to a higher secretion of CXCL12 as these results are in agreement with the differences in CXCL12 expression and secretion. It can be of prime importance as CXCL12-CXCR4 is a well-recognized axis crucial for the recruitment of different stem and progenitor cells, not only those from hematopoietic lineages. CXCL12 attracts mesenchymal stromal cells, endothelial progenitor cells, neural stem cells, smooth muscle progenitors, and fibroblast progenitor cells and therefore mediates the regeneration process in various tissues and organs [43]. It was shown that a local increase in CXCL12 concentration enhances also the healing of injured tendon. Shen et al. [44] demonstrated that CXCL12 incorporated into knitted silk-collagen sponge scaffold improved the efficacy of tendon regeneration by increasing the recruitment of fibroblast-like cells and tendon extracellular matrix production in comparison to the scaffold without CXCL12. More recently, the positive effect of CXCL12 on tendon regeneration was confirmed by Sun et al. [45]. In their study, the use of collagen scaffold with attached collagen-binding SDF-1 resulted in improved mechanical properties of regenerated tendons in comparison to the scaffold itself. Our results suggest that hBM-MSC Y can be more effective in enhancing tendon regeneration after local administration than hASCs because of more intensive CXCL12 production. On the other hand, the paracrine activity can be modified by many factors; therefore, it is difficult to predict if the difference in secretion would be retained in situ after transplantation. CXCL6 is the third chemokine which was analyzed more in detail in our study. In the case of this molecule, the microarray results were not confirmed by either RT-PCR or ELISA assays in which no significant differences were found between hBM-MSCs and hASCs.

In the research discussed here, we additionally evaluated the influence of BM donors' age on expression of chosen factors. Our results indicate that the impact of donor age on analyzed cell parameters is less significant than the influence of the cell source; however, BM-MSCs from elder donors seem to have higher tenogenic activity than do BM-MSCs from young donors. The literature data are not consistent in this issue. There are several studies in which authors conclude that the differentiation potential of MSCs is not significantly affected by donor age [18, 46]. On the other hand, some reports demonstrate that human MSCs isolated from aged donors present impaired therapeutic properties [47, 48]. In one study, BM-MSCs from young and aged donors underwent proteomic analysis after induction to tenogenic differentiation. It has been demonstrated that more than 200 proteins have a different expression in BM younger and elder donors. The differentially expressed proteins were associated with cell viability and death or antioxidant potential, as well as general protein metabolism, but not with tenogenic differentiation potential [49]. In our study, differences between the hBM-MSC Y and hBM-MSC A groups at the level of gene expression for chemokines were significant only for *CXCL12*. A higher *CXCL12* expression was in the hBM-MSC Y group. In the secretion activity test, the difference between cells from young and aged donors did not reach statistical significance, but the functional test was consistent with gene expression analysis. However, for SMAD3 and COL14A1, the differences between bone marrow groups were only significant at the protein level. Interestingly, in both cases, a higher protein level was observed for cells from aged bone marrow donors. For the other genes and proteins included in the analysis, there were no differences between the groups hBM-MSC Y and hBM-MSC A.

## 5. Conclusions

Cellular therapies are now being seriously considered for the treatment of tendon injuries. The possibility of using native MSCs as well as partially differentiating into the tenogenic pathway has its advantages and disadvantages. Depending on the type of injury and the decision between auto- or allogeneic transplant, it may be necessary to choose a different way of cell preparation. Regardless of the way chosen, the selection of a suitable source of cells seems to be crucial. In our study, we present the first comparison of human MSCs from the bone marrow and adipose tissue in the context of potential use in tendon therapy. What is important, the comparison concerned the base level of selected genes. This is important for two reasons. Firstly, it shows which cell source is more preferably selected for therapy with the use of nondifferentiated cells. Secondly, it allows reference to other studies where MSCs induced by various tenogenic factors or their combinations are tested. Such a comparison allows inferring whether the fold change for the expression of genes important for tenogenesis after induction is higher than for untreated BM-MSCs which still remain the gold standard for cellular therapies. Based on these results, we can suggest that for therapy of tendon injuries, MSCs originating from the bone marrow can be more beneficial than ASCs. The influence of the age of bone marrow donors seems to be less crucial than MSC source; however, cells from elder donors displayed a higher expression of some tenogenic markers than BM-MSCs from younger donors.

## Data Availability

The list of genes of significantly different expression between groups in microarray analysis is presented in Supplementary Information File 1. The Microarray Data used to support the findings of this study have been deposited in the NCBI's Gene Expression Omnibus and are accessible through GEO Series accession number GSE128949 (https://www.ncbi.nlm.nih.gov/geo/query/acc.cgi?acc=GSE128949). The flow cytometry, microarray, real-time qPCR, secretion, Western blot, and migration assay data used to support the findings of this study are available from the corresponding author upon request.

## Supplementary Materials

Supplementary Materials Supplementary material S1: images from automatic imaging reader Cytation™ 1. A, A', and A” present the same field of view. Cells migrating from the upper compartment of the inserts are stained with DID (red color). Cell nuclei are stained with DAPI (blue color). A' and A” demonstrate the method of analysis (A'—nuclei numbering, A”—migrating cells numbering), scale bar—300 *μ*m. Supplementary material S2: the table provides the list of genes which displayed a significantly different expression between hASCs and hBM-MSCs A based on microarray analysis. Supplementary material S3: the table provides the mean Δct and SEM values for all genes and all groups analyzed in this study (*n* = 8 in each group).
