# Supplementary material for: The Influence of Cell Source and Donor Age on the Tenogenic Potential and Chemokine Secretion of Human Mesenchymal Stromal Cells
Source: Stem Cells Int. 2019 May 7;2019:1613701. doi: 10.1155/2019/1613701 (PMC6530320; doi:10.1155/2019/1613701)
Supplement: Supplementary Materials — Supplementary material S1: images from automatic imaging reader Cytation™ 1. A, A', and A” present the same field of view. Cells migrating from the upper compartment of the inserts are stained with DID (red color). Cell nuclei are stained with DAPI (blue color). A' and A” demonstrate the method of analysis (A'—nuclei numbering, A”—migrating cells numbering), scale bar—300 μm. Supplementary material S2: the table provides the list of genes which displayed a significantly different expression between hASCs and hBM-MSCs A based on microarray analysis. Supplementary material S3: the table provides the mean Δct and SEM values for all genes and all groups analyzed in this study (n = 8 in each group). [file 1613701.f1.pdf]

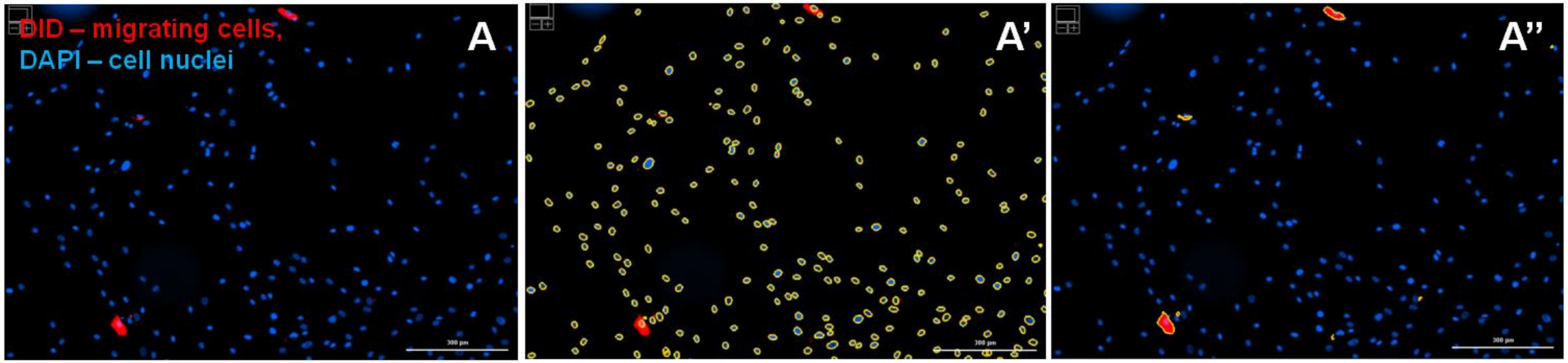

Supplementary material. S1. Images from automatic imaging reader Cytation™ 1. A, A’, A’’ present the same field of view. Cells migrating from the upper compartment of the inserts are stained with DID (red color), Cell nuclei are stained with DAPI (blue color). A’ and A’’ demonstrate the method of analysis (A’ – nuclei numbering, A’’ – migrating cells numbering), scale bar – 300 µm.

	ASC		BM-MSC Y		BM-MSC A	
	mean	SEM	mean	SEM	mean	SEM
CXCL6	2.240	0.727	4.158	0.677	2.149	1.816
CXCL16	4.277	0.248	0.763	0.405	1.130	0.346
CXCL12	-2.855	0.483	-4.528	0.226	-3.616	0.318
SMAD3	-0.511	0.191	-1.522	0.188	-1.332	0.215
TGFB2	3.742	0.533	1.238	0.210	0.990	0.568
COL14A	1.532	0.357	-0.397	0.736	-1.522	0.469
MHX	1.397	0.414	1.145	0.412	0.642	0.613
SCX	2.984	0.301	2.062	0.320	2.031	0.249
RUNX2	3.290	0.359	2.319	0.388	2.546	0.529
PPRAG	1.720	0.303	2.926	0.423	2.912	0.215

Supplementary material S3. The table provides the mean Δct and SEM values for all genes and all groups analyzed in this study (n=8 in each group).

Supplementary material S2. The table provides the list of genes which displayed significantly different expression between hASCs and hBM-MSCs A based on microarray

nr	Exp p-value	Exp Fold Change	ID	Symbol	Entrez Gene Name
1	2.84E-05	16.896	16960355	TM4SF1	transmembrane 4 L six family member 1
2	9.10E-09	12.238	16684080	IFI6	interferon alpha inducible protein 6
3	3.93E-08	11.851	16667702	VCAM1	vascular cell adhesion molecule 1
4	1.54E-05	9.944	16840113	CXCL16	C-X-C motif chemokine ligand 16
5	2.01E-06	9.123	16852463	RAB27B	RAB27B, member RAS oncogene family
6	2.55E-04	8.228	16971409	MAB21L2	mab-21 like 2
7	6.82E-05	8.100	17112918	BEX1	brain expressed X-linked 1
8	1.57E-05	7.722	16853879	PIEZO2	piezo type mechanosensitive ion channel component 2
9	3.28E-06	7.245	17004208	FOXC1	forkhead box C1
10	3.34E-04	6.584	16666509	IFI44	interferon induced protein 44
11	1.79E-04	6.264	16800630	C15orf48	chromosome 15 open reading frame 48
12	1.07E-04	6.142	16757373	OAS2	2'-5'-oligoadenylate synthetase 2
13	2.24E-04	6.001	17012859	PDE7B	phosphodiesterase 7B
14	9.65E-04	5.696	16925398	RUNX1-IT1	RUNX1 intronic transcript 1
15	1.73E-04	5.662	16666485	IFI44L	interferon induced protein 44 like
16	5.90E-04	5.486	16844558	KRTAP1-5	keratin associated protein 1-5
17	3.56E-02	5.438	16960304	CP	ceruloplasmin (ferroxidase)
18	6.10E-05	5.252	16909724	ARL4C	ADP ribosylation factor like GTPase 4C
19	3.04E-03	5.180	17117110	CD24	CD24 molecule
20	5.52E-04	5.142	16943241	COL8A1	collagen type VIII alpha 1
21	4.15E-02	5.066	16967779	CXCL6	C-X-C motif chemokine ligand 6
22	7.96E-05	4.940	17075553	STC1	stanniocalcin 1
23	4.15E-02	4.870	16698185	CHI3L1	chitinase 3 like 1
24	1.72E-03	4.798	16878947	LTBP1	latent transforming growth factor beta binding protein 1
25	1.35E-02	4.775	16767254	IL26	interleukin 26
26	2.08E-04	4.673	17080648	HAS2	hyaluronan synthase 2
27	3.83E-03	4.672	17051553	CPA4	carboxypeptidase A4
28	8.22E-05	4.551	16677556	TGFB2	transforming growth factor beta 2
29	1.23E-03	4.427	16952782	TMEM158	transmembrane protein 158 (gene/pseudogene)
30	1.04E-02	4.412	16886491	TNFAIP6	TNF alpha induced protein 6
31	3.76E-04	4.412	16928204	POM121L9P	POM121 transmembrane nucleoporin like 9, pseudogene
32	2.38E-04	4.368	17012148	GJA1	gap junction protein alpha 1

33	6.41E-04	4.318	16791319	ADCY4	adenylate cyclase 4
34	4.21E-04	4.317	16852179	SLC14A1	solute carrier family 14 member 1 (Kidd blood group)
35	6.92E-04	4.266	17075112	PSD3	pleckstrin and Sec7 domain containing 3
36	4.87E-03	4.245	16676167	PRELP	proline and arginine rich end leucine rich repeat protein
37	6.70E-04	4.238	16793674	LOC105370526	uncharacterized LOC105370526
38	1.42E-05	4.214	16911338	PLCB4	phospholipase C beta 4
39	1.22E-02	4.176	16913537	LBP	lipopolysaccharide binding protein
40	3.57E-03	4.134	16785897	SMOC1	SPARC related modular calcium binding 1
41	1.06E-04	4.123	17012946	TNFAIP3	TNF alpha induced protein 3
42	1.53E-03	4.100	16681304	ERRFI1	ERBB receptor feedback inhibitor 1
43	2.24E-03	4.030	16911493	SPTLC3	serine palmitoyltransferase long chain base subunit 3
44	2.40E-04	4.012	16851933	GALNT1	polypeptide N-acetylgalactosaminyltransferase 1
45	2.20E-03	3.943	16705507	SRGN	serglycin
46	5.54E-03	3.901	17072162	COL14A1	collagen type XIV alpha 1 chain
47	5.35E-04	3.898	16810341	CA12	carbonic anhydrase 12
48	1.06E-03	3.892	17105332	SRPX2	sushi repeat containing protein, X-linked 2
49	1.37E-03	3.873	16698949	KCNH1	potassium voltage-gated channel subfamily H member 1
50	1.34E-02	3.829	16947715	SERPINI1	serpin family I member 1
51	3.28E-03	3.828	16852573	NEDD4L	neural precursor cell expressed, E3 ubiquitin protein ligase
52	6.70E-03	3.806	17087109	C9orf3	chromosome 9 open reading frame 3
53	1.03E-04	3.720	16904365	IFIH1	interferon induced with helicase C domain 1
54	9.35E-03	3.714	17023799	SLC2A12	solute carrier family 2 member 12
55	6.84E-03	3.695	16793129	BMP4	bone morphogenetic protein 4
56	4.03E-02	3.684	16716659	RBP4	retinol binding protein 4
57	4.39E-05	3.684	16720085	IFITM1	interferon induced transmembrane protein 1
58	1.07E-04	3.658	16983765	NPR3	natriuretic peptide receptor 3
59	8.61E-04	3.651	16998059	ARRDC3	arrestin domain containing 3
60	1.40E-03	3.611	16713530	CXCL12	C-X-C motif chemokine ligand 12
61	2.04E-02	3.605	16851230	ANKRD20A5P	ankyrin repeat domain 20 family member A5, pseudogene
62	4.06E-02	3.590	16833204	CCL2	C-C motif chemokine ligand 2
63	2.61E-03	3.579	17095056	PRUNE2	prune homolog 2
64	7.94E-03	3.519	16675197	PLA2G4A	phospholipase A2 group IVA
65	2.51E-02	3.485	16776936	TMEM255B	transmembrane protein 255B
66	1.71E-03	3.482	17050591	MET	MET proto-oncogene, receptor tyrosine kinase
67	1.63E-02	3.459	16800680	SQRDL	sulfide quinone reductase-like (yeast)
68	7.56E-04	3.394	17043856	AHR	aryl hydrocarbon receptor

69	1.80E-03	3.379	16984287	PTGER4	prostaglandin E receptor 4
70	1.05E-02	3.315	16911261	BMP2	bone morphogenetic protein 2
71	1.31E-03	3.314	16699533	DUSP10	dual specificity phosphatase 10
72	3.73E-03	3.298	17012632	ENPP1	ectonucleotide pyrophosphatase/phosphodiesterase 1
73	2.06E-03	3.273	16956792	ABI3BP	ABI family member 3 binding protein
74	2.62E-04	3.253	17067031	DOCK5	dedicator of cytokinesis 5
75	4.23E-03	3.252	16744616	CADM1	cell adhesion molecule 1
76	4.26E-03	3.251	16951696	NEK10	NIMA related kinase 10
77	3.49E-02	3.248	16975015	CCKAR	cholecystokinin A receptor
78	3.09E-04	3.245	17002898	STC2	stanniocalcin 2
79	1.01E-04	3.223	16968735	HERC6	HECT and RLD domain containing E3 ubiquitin protein ligase family member 6
80	8.87E-03	3.205	17043982	ITGB8	integrin subunit beta 8
81	5.70E-03	3.158	17088100	UGCG	UDP-glucose ceramide glucosyltransferase
82	1.55E-02	3.156	17080082	ANGPT1	angiopoietin 1
83	1.61E-04	3.135	16834091	IGFBP4	insulin like growth factor binding protein 4
84	4.22E-03	3.129	17012721	EYA4	EYA transcriptional coactivator and phosphatase 4
85	2.93E-04	3.125	16984730	FST	follistatin
86	1.57E-03	3.118	16952769	CDCP1	CUB domain containing protein 1
87	8.92E-04	3.109	17093090	DDX58	DEXD/H-box helicase 58
88	1.14E-03	3.093	16907488	RAPH1	Ras association (RalGDS/AF-6) and pleckstrin homology domains 1
89	5.27E-04	3.088	17080666	LOC105375734	uncharacterized LOC105375734
90	9.44E-05	3.085	16982183	SORBS2	sorbin and SH3 domain containing 2
91	2.38E-03	3.077	16937943	SLC6A6	solute carrier family 6 member 6
92	2.79E-03	3.051	16718670	GFRA1	GDNF family receptor alpha 1
93	7.12E-03	3.046	16867784	C3	complement component 3
94	3.31E-03	3.043	16690704	SLC16A4	solute carrier family 16 member 4
95	9.64E-05	3.037	16794846	PGF	placental growth factor
96	3.47E-03	3.026	16714618	ANK3	ankyrin 3, node of Ranvier (ankyrin G)
97	1.24E-02	3.024	17012392	RSPO3	R-spondin 3
98	1.87E-02	3.010	17077826	MYBL1	MYB proto-oncogene like 1
99	1.76E-03	3.009	16914084	WISP2	WNT1 inducible signaling pathway protein 2
100	2.04E-04	3.004	17059195	CACNA2D1	calcium voltage-gated channel auxiliary subunit alpha2delta 1
101	4.28E-02	2.987	16728384	mir-548	microRNA 579
102	2.98E-02	2.984	16751190	METTL7A	methyltransferase like 7A
103	2.35E-02	2.975	17048072	STEAP1	six transmembrane epithelial antigen of the prostate 1
104	2.00E-03	2.969	16690566	SORT1	sortilin 1

105	7.31E-03	2.953	16944695	PARP14	poly(ADP-ribose) polymerase family member 14
106	1.06E-04	2.944	16802251	SMAD3	SMAD family member 3
107	1.09E-02	2.943	16670469	C1orf54	chromosome 1 open reading frame 54
108	1.45E-02	2.935	16962632	P3H2	prolyl 3-hydroxylase 2
109	1.25E-02	2.932	16940055	CLEC3B	C-type lectin domain family 3 member B
110	2.43E-03	2.922	16838330	SYNGR2	synaptogyrin 2
111	6.10E-03	2.921	16689354	GBP2	guanylate binding protein 2
112	8.20E-03	2.910	16712879	SVIL	supervillin
113	1.20E-02	2.893	16906308	TFPI	tissue factor pathway inhibitor
114	3.23E-04	2.874	16923031	MX1	MX dynamin like GTPase 1
115	2.66E-02	2.870	17096958	FRRS1L	ferric chelate reductase 1 like
116	2.15E-03	2.863	17061881	DOCK4	dedicator of cytokinesis 4
117	1.19E-03	2.858	16719217	CHST15	carbohydrate (N-acetylgalactosamine 4-sulfate 6-O) sulfotransferase 15
118	3.59E-04	2.850	16914478	EYA2	EYA transcriptional coactivator and phosphatase 2
119	1.89E-02	2.848	16938133	GALNT15	polypeptide N-acetylgalactosaminyltransferase 15
120	9.02E-03	2.845	16967771	CXCL8	C-X-C motif chemokine ligand 8
121	1.23E-02	2.839	17088116	MIR4668	microRNA 4668
122	6.17E-03	2.824	16712934	mir-604	microRNA 604
123	8.98E-04	2.811	16819252	MT1F	metallothionein 1F
124	5.77E-03	2.809	17014114	SYNJ2	synaptojanin 2
125	1.58E-02	2.806	16697370	PTGS2	prostaglandin-endoperoxide synthase 2
126	1.03E-02	2.804	16925594	LINC01423	long intergenic non-protein coding RNA 1423
127	9.55E-03	2.802	16730522	BIRC3	baculoviral IAP repeat containing 3
128	3.64E-02	2.799	17014257	FNDC1	fibronectin type III domain containing 1
129	3.42E-03	2.793	16665717	LEPR	leptin receptor
130	1.88E-03	2.787	16933760	LIF	leukemia inhibitory factor
131	3.56E-03	2.751	16766279	SNORD59B	small nucleolar RNA, C/D box 59B
132	1.17E-02	2.745	17080342	TRPS1	transcriptional repressor GATA binding 1
133	7.25E-03	2.744	16979339	PDE5A	phosphodiesterase 5A
134	1.56E-02	2.744	17063221	FAM180A	family with sequence similarity 180 member A
135	6.78E-03	2.735	16819152	CES1P1	carboxylesterase 1 pseudogene 1
136	4.22E-04	2.730	16688506	NEGR1	neuronal growth regulator 1
137	4.22E-03	2.697	16957095	CD47	CD47 molecule
138	3.78E-04	2.694	16657594	ISG15	ISG15 ubiquitin-like modifier
139	2.19E-04	2.690	16955939	LRIG1	leucine rich repeats and immunoglobulin like domains 1
140	3.64E-03	2.687	17043529	C1GALT1	core 1 synthase, glycoprotein-N-acetylgalactosamine 3-beta-galactosyltransferase 1

141	8.12E-03	2.681	16786010	SIPA1L1	signal induced proliferation associated 1 like 1
142	5.30E-04	2.679	16678095	LOC102723834	uncharacterized LOC102723834
143	1.52E-02	2.677	16661687	EPB41	erythrocyte membrane protein band 4.1
144	4.75E-03	2.665	16885874	MGAT5	mannosyl (alpha-1,6-)-glycoprotein beta-1,6-N-acetyl-glucosaminyltransferase
145	3.65E-03	2.658	16886717	GALNT5	polypeptide N-acetylgalactosaminyltransferase 5
146	1.34E-02	2.657	17051860	BPGM	bisphosphoglycerate mutase
147	2.23E-02	2.647	16755908	DRAM1	DNA damage regulated autophagy modulator 1
148	1.39E-02	2.641	16985688	MARVELD2	MARVEL domain containing 2
149	2.70E-03	2.630	16896561	CYP1B1	cytochrome P450 family 1 subfamily B member 1
150	3.16E-03	2.628	17005573	HIST1H2BD	histone cluster 1, H2bd
151	3.93E-03	2.624	16941024	SEMA3B	semaphorin 3B
152	5.74E-03	2.621	16757347	OAS3	2'-5'-oligoadenylate synthetase 3
153	3.38E-03	2.616	17076412	LOC102723716	uncharacterized LOC102723716
154	1.66E-05	2.608	17063005	PLXNA4	plexin A4
155	2.53E-03	2.602	16676355	NFASC	neurofascin
156	5.63E-03	2.599	16938724	FBXL2	F-box and leucine rich repeat protein 2
157	1.05E-02	2.598	17096904	CTNNAL1	catenin alpha like 1
158	7.52E-05	2.596	17101873	LOC101928389	uncharacterized LOC101928389
159	1.70E-02	2.589	16802232	SMAD6	SMAD family member 6
160	6.81E-03	2.589	16683445	FUCA1	fucosidase, alpha-L- 1, tissue
161	1.97E-02	2.587	16854268	NPC1	NPC intracellular cholesterol transporter 1
162	2.31E-02	2.572	16692834	CTSS	cathepsin S
163	5.53E-03	2.570	16884918	INSIG2	insulin induced gene 2
164	1.39E-02	2.570	17086634	CKS2	CDC28 protein kinase regulatory subunit 2
165	1.57E-02	2.570	17006949	CFB	complement factor B
166	1.55E-02	2.569	16840122	VMO1	vitelline membrane outer layer 1 homolog
167	2.60E-03	2.562	16671874	SYT11	synaptotagmin 11
168	7.57E-03	2.558	16885135	INHBB	inhibin beta B subunit
169	1.20E-02	2.549	16906571	STAT4	signal transducer and activator of transcription 4
170	1.60E-03	2.540	16824352	XYLT1	xylosyltransferase 1
171	1.05E-03	2.538	16906733	STK17B	serine/threonine kinase 17b
172	1.07E-02	2.537	16958124	PARP9	poly(ADP-ribose) polymerase family member 9
173	1.06E-02	2.536	16809263	DMXL2	Dmx like 2
174	2.40E-02	2.534	16714135	ERCC6	ERCC excision repair 6, chromatin remodeling factor
175	1.59E-02	2.528	16762759	TMTC1	transmembrane and tetratricopeptide repeat containing 1
176	1.09E-02	2.523	16986138	ARHGEF28	Rho guanine nucleotide exchange factor 28

177	1.05E-02	2.521	16778938	HTR2A	5-hydroxytryptamine receptor 2A
178	1.78E-02	2.520	17092115	GLIS3	GLIS family zinc finger 3
179	1.35E-03	2.519	16703251	KIAA1217	KIAA1217
180	1.22E-02	2.510	16718666	ABLIM1	actin binding LIM protein 1
181	2.66E-02	2.502	16821562	KIAA0513	KIAA0513
182	1.34E-02	2.495	16702007	AKR1C3	aldo-keto reductase family 1, member C3
183	1.01E-02	2.492	16967794	CXCL1	C-X-C motif chemokine ligand 1
184	4.82E-03	2.487	16907044	SATB2	SATB homeobox 2
185	1.88E-04	2.485	16979163	ARSJ	arylsulfatase family member J
186	5.36E-03	2.480	16869427	CACNA1A	calcium voltage-gated channel subunit alpha1 A
187	2.28E-02	2.477	16978976	CFI	complement factor I
188	7.93E-04	2.472	16919022	SAMHD1	SAM and HD domain containing deoxynucleoside triphosphate triphosphohydrolase 1
189	7.67E-03	2.465	16738897	SLC15A3	solute carrier family 15 member 3
190	2.14E-02	2.463	16769159	GNPTAB	N-acetylglucosamine-1-phosphate transferase alpha and beta subunits
191	1.14E-02	2.458	16830577	CD68	CD68 molecule
192	4.57E-03	2.454	16984056	SLC1A3	solute carrier family 1 member 3
193	6.90E-03	2.448	16721585	OLFML1	olfactomedin like 1
194	4.15E-02	2.447	16808306	PPIP5K1	diphosphoinositol pentakisphosphate kinase 1
195	2.06E-03	2.447	16723546	CAT	catalase
196	4.87E-03	2.445	17051159	HILPDA	hypoxia inducible lipid droplet associated
197	1.75E-02	2.444	17110367	mir-221	microRNA 221
198	4.11E-03	2.441	17057525	TNS3	tensin 3
199	2.76E-03	2.439	17060143	BAIAP2L1	BAI1 associated protein 2 like 1
200	3.34E-02	2.438	16715793	KCNMA1	potassium calcium-activated channel subfamily M alpha 1
201	3.85E-03	2.435	16917183	JAG1	jagged 1
202	2.07E-02	2.422	17014091	SNX9	sorting nexin 9
203	1.07E-02	2.417	17020464	RAB23	RAB23, member RAS oncogene family
204	2.15E-02	2.414	16986065	TMEM171	transmembrane protein 171
205	2.20E-02	2.407	16748496	APOLD1	apolipoprotein L domain containing 1
206	1.59E-02	2.404	17010760	NT5E	5'-nucleotidase ecto
207	3.17E-02	2.401	16855600	CCBE1	collagen and calcium binding EGF domains 1
208	6.46E-03	2.399	16700806	LYST	lysosomal trafficking regulator
209	3.85E-03	2.399	16781285	GAS6	growth arrest specific 6
210	8.38E-03	2.397	16675398	CFH	complement factor H
211	2.18E-02	2.394	17021596	RRAGD	Ras related GTP binding D
212	4.30E-03	2.394	16782826	NFATC4	nuclear factor of activated T-cells 4

213	2.04E-03	2.392	16835672	ITGA3	integrin subunit alpha 3
214	1.19E-03	2.390	17086208	TLE4	transducin like enhancer of split 4
215	4.73E-03	2.390	17020823	SLC17A5	solute carrier family 17 member 5
216	2.47E-02	2.388	16919567	SDC4	syndecan 4
217	2.42E-02	2.388	17060049	DLX5	distal-less homeobox 5
218	6.47E-03	2.382	16975890	OCIAD2	OCIA domain containing 2
219	3.51E-03	2.380	16754373	GLIPR1	GLI pathogenesis related 1
220	4.76E-02	2.374	17050797	CPED1	cadherin like and PC-esterase domain containing 1
221	2.09E-02	2.373	16815807	ATF7IP2	activating transcription factor 7 interacting protein 2
222	2.08E-02	2.364	17089003	OLFML2A	olfactomedin like 2A
223	1.41E-02	2.361	16700872	LYST	lysosomal trafficking regulator
224	2.62E-02	2.359	16806014	HERC2P2	hect domain and RLD 2 pseudogene 2
225	3.97E-03	2.359	16678579	GALNT2	polypeptide N-acetylgalactosaminyltransferase 2
226	7.60E-03	2.358	16924602	ADAMTS1	ADAM metallopeptidase with thrombospondin type 1 motif 1
227	6.40E-03	2.357	16768406	LUM	lumican
228	2.36E-02	2.357	17097797	BRINP1	BMP/retinoic acid inducible neural specific 1
229	2.67E-02	2.356	16832147	ALDH3A2	aldehyde dehydrogenase 3 family member A2
230	9.94E-03	2.355	16695741	OLFML2B	olfactomedin like 2B
231	1.00E-02	2.353	17022670	TRAF3IP2	TRAF3 interacting protein 2
232	3.86E-03	2.351	16965680	TBC1D19	TBC1 domain family member 19
233	4.52E-02	2.351	16922495	CBR3	carbonyl reductase 3
234	5.38E-03	2.348	16851486	LAMA3	laminin subunit alpha 3
235	3.77E-02	2.348	16749782	FGD4	FYVE, RhoGEF and PH domain containing 4
236	1.10E-02	2.345	17066601	SLC39A14	solute carrier family 39 member 14
237	1.07E-02	2.345	16819217	MT1E	metallothionein 1E
238	3.53E-03	2.339	16980946	PDGFC	platelet derived growth factor C
239	3.27E-03	2.337	16688339	WLS	wntless Wnt ligand secretion mediator
240	2.23E-03	2.333	16717235	AVPI1	arginine vasopressin induced 1
241	1.09E-02	2.332	17023414	PTPRK	protein tyrosine phosphatase, receptor type K
242	6.28E-04	2.329	16819224	MT1M	metallothionein 1M
243	1.21E-02	2.325	16762597	TM7SF3	transmembrane 7 superfamily member 3
244	2.61E-02	2.322	17025937	PHF10	PHD finger protein 10
245	3.70E-02	2.312	16955197	WNT5A	Wnt family member 5A
246	1.29E-02	2.312	16969009	PDLIM5	PDZ and LIM domain 5
247	2.03E-02	2.298	16951756	SLC4A7	solute carrier family 4 member 7
248	5.89E-03	2.293	16757324	OAS1	2'-5'-oligoadenylate synthetase 1

249	4.65E-03	2.293	16962661	CLDN1	claudin 1
250	3.17E-03	2.290	16782687	IRF9	interferon regulatory factor 9
251	1.20E-02	2.289	16818842	CYLD	CYLD lysine 63 deubiquitinase
252	1.03E-02	2.286	16935195	CBX6	chromobox 6
253	4.06E-03	2.276	16779766	KLF12	Kruppel like factor 12
254	1.14E-02	2.270	16731084	SIK2	salt inducible kinase 2
255	1.36E-03	2.270	17068139	PLEKHA2	pleckstrin homology domain containing A2
256	3.41E-03	2.270	16836021	ABCC3	ATP binding cassette subfamily C member 3
257	2.17E-02	2.268	17018652	MIR3925	microRNA 3925
258	3.44E-02	2.268	16970536	HSPA4L	heat shock protein family A (Hsp70) member 4 like
259	2.21E-03	2.268	16905590	FKBP7	FK506 binding protein 7
260	4.76E-03	2.264	16712655	ANKRD26	ankyrin repeat domain 26
261	8.03E-03	2.261	16990267	PCDHB5	protocadherin beta 5
262	3.64E-02	2.258	16758671	TCTN2	tectonic family member 2
263	8.72E-04	2.251	16922396	MRPS6	mitochondrial ribosomal protein S6
264	1.80E-02	2.248	17010941	PM20D2	peptidase M20 domain containing 2
265	1.33E-02	2.247	16889958	ZDBF2	zinc finger DBF-type containing 2
266	3.96E-02	2.244	16906000	SESTD1	SEC14 and spectrin domain containing 1
267	1.26E-02	2.242	16998682	EFNA5	ephrin A5
268	2.75E-03	2.237	16960186	PLSCR1	phospholipid scramblase 1
269	2.92E-02	2.236	16660933	LDLRAP1	low density lipoprotein receptor adaptor protein 1
270	3.32E-02	2.235	16820388	PLA2G15	phospholipase A2 group XV
271	4.74E-02	2.229	17086386	NTRK2	neurotrophic receptor tyrosine kinase 2
272	2.36E-02	2.215	17045291	EPDR1	ependymin related 1
273	9.02E-04	2.214	16765697	ITGA7	integrin subunit alpha 7
274	7.18E-04	2.213	17059932	PON2	paraoxonase 2
275	2.43E-02	2.213	16739911	NRXN2	neurexin 2
276	1.59E-04	2.209	16998906	MCC	mutated in colorectal cancers
277	1.63E-02	2.207	17111918	ZMYM3	zinc finger MYM-type containing 3
278	1.21E-02	2.206	16841660	CDRT1	CMT1A duplicated region transcript 1
279	2.78E-02	2.205	16669781	LOC100996763/NOTCH2NL	notch 2 N-terminal like
280	2.78E-02	2.203	16766093	STAT2	signal transducer and activator of transcription 2
281	1.24E-02	2.198	17058826	HIP1	huntingtin interacting protein 1
282	2.05E-02	2.197	17091554	UAP1L1	UDP-N-acetylglucosamine pyrophosphorylase 1 like 1
283	7.75E-03	2.195	17079808	RRM2B	ribonucleotide reductase regulatory TP53 inducible subunit M2B

284	2.85E-02	2.195	16855510	ATP8B1	ATPase phospholipid transporting 8B1
285	3.44E-04	2.189	17087057	C9orf3	chromosome 9 open reading frame 3
286	2.27E-03	2.186	17015033	GMDS	GDP-mannose 4,6-dehydratase
287	6.57E-03	2.186	16705089	BICC1	BicC family RNA binding protein 1
288	1.24E-04	2.185	17109042	MID1	midline 1
289	2.35E-03	2.184	16665796	SGIP1	SH3 domain GRB2 like endophilin interacting protein 1
290	5.85E-03	2.183	16859711	MAST3	microtubule associated serine/threonine kinase 3
291	2.20E-02	2.182	17001299	DPYSL3	dihydropyrimidinase like 3
292	2.04E-02	2.178	16819994	HSF4	heat shock transcription factor 4
293	2.11E-02	2.177	16691314	TSPAN2	tetraspanin 2
294	1.13E-03	2.168	16984562	PARP8	poly(ADP-ribose) polymerase family member 8
295	1.67E-02	2.166	17022691	FYN	FYN proto-oncogene, Src family tyrosine kinase
296	8.75E-03	2.163	16772042	CCDC92	coiled-coil domain containing 92
297	1.76E-02	2.160	16946787	HPS3	HPS3, biogenesis of lysosomal organelles complex 2 subunit 1
298	1.16E-02	2.159	16821541	CRISPLD2	cysteine rich secretory protein LCCL domain containing 2
299	2.27E-02	2.158	17014295	WTAP	Wilms tumor 1 associated protein
300	2.86E-02	2.153	16693082	SELENBP1	selenium binding protein 1
301	1.49E-02	2.152	16805594	MEF2A	myocyte enhancer factor 2A
302	2.14E-02	2.151	17022623	REV3L	REV3 like, DNA directed polymerase zeta catalytic subunit
303	3.23E-02	2.150	16741334	MRGPRF	MAS related GPR family member F
304	2.81E-02	2.149	17097869	MEGF9	multiple EGF like domains 9
305	2.11E-02	2.149	16891082	CYP27A1	cytochrome P450 family 27 subfamily A member 1
306	1.48E-02	2.146	16934671	TST	thiosulfate sulfurtransferase
307	8.65E-03	2.146	16846864	MMD	monocyte to macrophage differentiation associated
308	5.08E-03	2.146	16852473	mir-4529	microRNA 4529
309	5.56E-04	2.146	17078134	EYA1	EYA transcriptional coactivator and phosphatase 1
310	4.84E-02	2.146	16730967	C11orf87	chromosome 11 open reading frame 87
311	1.84E-02	2.145	17102210	PDK3	pyruvate dehydrogenase kinase 3
312	3.93E-02	2.140	16848070	SLC16A6	solute carrier family 16 member 6
313	2.30E-02	2.140	16726876	NEAT1	nuclear paraspeckle assembly transcript 1 (non-protein coding)
314	2.66E-02	2.139	16840723	SAT2	spermidine/spermine N1-acetyltransferase family member 2
315	7.40E-03	2.139	16692632	HIST2H2BE	histone cluster 2, H2be
316	1.37E-02	2.139	16665621	CACHD1	cache domain containing 1
317	1.42E-02	2.138	16854856	EPG5	ectopic P-granules autophagy protein 5 homolog
318	7.18E-03	2.131	17080486	TNFRSF11B	TNF receptor superfamily member 11b
319	2.35E-02	2.130	17063461	HIPK2	homeodomain interacting protein kinase 2

320	1.35E-02	2.130	16956714	DCBLD2	discoidin, CUB and LCCL domain containing 2
321	9.07E-03	2.123	16740914	PC	pyruvate carboxylase
322	3.02E-02	2.119	17097211	PTGR1	prostaglandin reductase 1
323	6.25E-03	2.118	16906534	STAT1	signal transducer and activator of transcription 1
324	1.81E-02	2.118	17025267	SOD2	superoxide dismutase 2, mitochondrial
325	1.52E-02	2.118	16911418	SNAP25	synaptosome associated protein 25
326	2.39E-02	2.118	16758075	P2RX4	purinergic receptor P2X 4
327	2.51E-03	2.117	16766822	LRIG3	leucine rich repeats and immunoglobulin like domains 3
328	2.71E-02	2.115	16877297	TRIB2	tribbles pseudokinase 2
329	3.42E-02	2.114	16914859	mir-645	microRNA 645
330	7.16E-03	2.114	17110372	LOC401585	uncharacterized LOC401585
331	1.25E-03	2.114	16731068	LAYN	layilin
332	1.92E-02	2.111	16739026	CYB561A3	cytochrome b561 family member A3
333	1.23E-03	2.107	16917689	RALGAPA2	Ral GTPase activating protein catalytic alpha subunit 2
334	3.94E-02	2.106	16994618	MYO10	myosin X
335	6.67E-03	2.104	16833171	TMEM98	transmembrane protein 98
336	8.85E-03	2.102	16981730	VEGFC	vascular endothelial growth factor C
337	3.57E-03	2.102	16699904	ENAH	enabled homolog (Drosophila)
338	3.23E-02	2.101	16808308	PPIP5K1	diphosphoinositol pentakisphosphate kinase 1
339	1.52E-02	2.101	17022849	LINC01268	long intergenic non-protein coding RNA 1268
340	2.28E-02	2.100	16880168	RPL23AP32	ribosomal protein L23a pseudogene 32
341	5.01E-03	2.098	16916920	SLC23A2	solute carrier family 23 member 2
342	1.38E-02	2.098	17075973	DUSP4	dual specificity phosphatase 4
343	1.33E-02	2.097	16918953	NDRG3	NDRG family member 3
344	2.58E-02	2.095	16714890	HERC4	HECT and RLD domain containing E3 ubiquitin protein ligase 4
345	1.20E-02	2.094	17110586	SLC38A5	solute carrier family 38 member 5
346	3.34E-02	2.094	16996433	PLK2	polo like kinase 2
347	3.40E-03	2.094	17063977	LOC154761	family with sequence similarity 115, member C pseudogene
348	7.39E-03	2.093	16727232	CST6	cystatin E/M
349	3.33E-02	2.091	17007307	PSMB9	proteasome subunit beta 9
350	1.88E-02	2.091	16986203	HEXB	hexosaminidase subunit beta
351	2.46E-03	2.088	16881838	HK2	hexokinase 2
352	4.28E-02	2.088	17103653	GAGE1 (includes others)	G antigen 1
353	3.00E-02	2.086	16977094	SCARB2	scavenger receptor class B member 2
354	1.82E-02	2.086	16961551	PLD1	phospholipase D1
355	3.89E-02	2.085	16904152	PLA2R1	phospholipase A2 receptor 1

356	9.71E-03	2.084	16851022	CHMP1B	charged multivesicular body protein 1B
357	2.94E-02	2.083	16665165	LOC105378737	uncharacterized LOC105378737
358	2.61E-02	2.082	16954217	UBA7	ubiquitin like modifier activating enzyme 7
359	7.58E-04	2.081	16778559	EPSTI1	epithelial stromal interaction 1 (breast)
360	8.95E-03	2.080	16900635	SEMA4C	semaphorin 4C
361	3.85E-02	2.080	16918722	GDF5	growth differentiation factor 5
362	5.25E-03	2.077	16844572	KRTAP1-1	keratin associated protein 1-1
363	3.97E-02	2.076	16937579	IRAK2	interleukin 1 receptor associated kinase 2
364	4.12E-03	2.076	16820528	HAS3	hyaluronan synthase 3
365	5.33E-03	2.076	16734313	CTSD	cathepsin D
366	7.84E-03	2.073	16978793	PAPSS1	3'-phosphoadenosine 5'-phosphosulfate synthase 1
367	3.49E-02	2.073	16999147	DTWD2	DTW domain containing 2
368	2.13E-02	2.073	16944096	ATP6V1A	ATPase H+ transporting V1 subunit A
369	7.57E-03	2.072	16887062	SCN2A	sodium voltage-gated channel alpha subunit 2
370	1.11E-03	2.072	17059776	SAMD9L	sterile alpha motif domain containing 9 like
371	4.69E-02	2.071	16747270	TAPBPL	TAP binding protein like
372	3.47E-03	2.071	16944665	DTX3L	deltex E3 ubiquitin ligase 3L
373	3.21E-02	2.071	17042984	CHST12	carbohydrate (chondroitin 4) sulfotransferase 12
374	2.10E-03	2.068	16972993	TLR3	toll like receptor 3
375	4.72E-02	2.068	16780885	FAM155A	family with sequence similarity 155 member A
376	1.73E-02	2.068	17013773	AKAP12	A-kinase anchoring protein 12
377	2.51E-02	2.059	16836375	MSI2	musashi RNA binding protein 2
378	3.54E-02	2.057	16795368	STON2	stonin 2
379	3.29E-02	2.055	17051626	MEST	mesoderm specific transcript
380	3.12E-02	2.054	16841696	ZSWIM7	zinc finger SWIM-type containing 7
381	3.06E-02	2.054	17051441	STRIP2	striatin interacting protein 2
382	4.45E-02	2.052	16833420	CCL4	C-C motif chemokine ligand 4
383	1.16E-02	2.048	16669888	ANKRD35	ankyrin repeat domain 35
384	1.94E-02	2.047	16772811	ZNF605	zinc finger protein 605
385	1.09E-02	2.046	17013954	TIAM2	T-cell lymphoma invasion and metastasis 2
386	6.52E-03	2.046	17112769	GLA	galactosidase alpha
387	2.03E-02	2.043	16978995	ELOVL6	ELOVL fatty acid elongase 6
388	5.91E-03	2.042	16743517	MAML2	mastermind like transcriptional coactivator 2
389	2.44E-02	2.041	16711037	DIP2C	disco interacting protein 2 homolog C
390	1.12E-02	2.040	16771998	RILPL2	Rab interacting lysosomal protein like 2
391	4.90E-02	2.039	16807905	ZNF106	zinc finger protein 106

392	2.93E-02	2.039	16827679	NQO1	NAD(P)H quinone dehydrogenase 1
393	1.96E-02	2.038	17051152	LEP	leptin
394	4.56E-02	2.038	17055804	FAM126A	family with sequence similarity 126 member A
395	1.70E-02	2.035	16854702	INO80C	INO80 complex subunit C
396	3.02E-03	2.034	16983907	BRIX1	BRX1, biogenesis of ribosomes
397	2.96E-02	2.032	17022996	ROS1	ROS proto-oncogene 1, receptor tyrosine kinase
398	1.24E-02	2.030	17014064	ZDHHC14	zinc finger DHHC-type containing 14
399	2.82E-02	2.029	16688937	CTBS	chitobiase
400	3.90E-02	2.027	16741287	CPT1A	carnitine palmitoyltransferase 1A
401	3.23E-02	2.026	16987766	C5orf30	chromosome 5 open reading frame 30
402	1.27E-03	2.020	16841481	MIR1269B	microRNA 1269b
403	3.64E-02	2.019	16788036	BDKRB1	bradykinin receptor B1
404	2.24E-02	2.018	16900160	IGKV2-29	immunoglobulin kappa variable 2-29 (gene/pseudogene)
405	1.54E-02	2.017	16725405	TMEM132A	transmembrane protein 132A
406	3.27E-02	2.017	16792859	PYGL	phosphorylase, glycogen, liver
407	1.96E-02	2.016	17055191	RPA3	replication protein A3
408	3.09E-02	2.016	17003558	CLK4	CDC like kinase 4
409	2.24E-02	2.015	16828577	MAF	MAF bZIP transcription factor
410	2.27E-02	2.011	16967631	SLC4A4	solute carrier family 4 member 4
411	3.10E-02	2.010	17110365	mir-221	microRNA 221
412	7.23E-03	2.009	16739208	FTH1	ferritin heavy chain 1
413	1.95E-02	2.007	16908711	TUBA4A	tubulin alpha 4a
414	1.76E-02	2.007	16702175	PFKFB3	6-phosphofructo-2-kinase/fructose-2,6-biphosphatase 3
415	5.65E-03	2.007	16834545	IFI35	interferon induced protein 35
416	4.64E-02	2.006	17074795	LOC729732	uncharacterized LOC729732
417	1.09E-02	2.005	16669783	LOC100996763/NOTCH2NL	notch 2 N-terminal like
418	1.09E-03	2.004	16972249	PALLD	palladin, cytoskeletal associated protein
419	4.65E-02	2.002	17087098	mir-2278	microRNA 2278
420	1.63E-02	2.000	16728261	CCND1	cyclin D1
421	9.21E-03	1.998	17063480	PARP12	poly(ADP-ribose) polymerase family member 12
422	1.83E-02	1.998	16668420	CSF1	colony stimulating factor 1
423	5.66E-03	1.998	17022139	BVES	blood vessel epicardial substance
424	1.21E-02	1.997	16869350	DNASE2	deoxyribonuclease II, lysosomal
425	3.01E-02	1.996	17090190	NCS1	neuronal calcium sensor 1
426	3.88E-02	1.995	17105665	LINC00630	long intergenic non-protein coding RNA 630

427	2.91E-02	1.994	17059771	SAMD9	sterile alpha motif domain containing 9
428	3.07E-02	1.994	16839294	ABR	active BCR-related
429	4.71E-02	1.990	16973708	MXD4	MAX dimerization protein 4
430	1.20E-02	1.988	16975918	SGCB	sarcoglycan beta
431	2.21E-02	1.988	16708468	BTRC	beta-transducin repeat containing E3 ubiquitin protein ligase
432	1.35E-02	1.986	17047138	GTF2IRD1	GTF2I repeat domain containing 1
433	3.46E-03	1.986	16660103	ARHGEF10L	Rho guanine nucleotide exchange factor 10 like
434	4.33E-02	1.984	16670081	CHD1L	chromodomain helicase DNA binding protein 1 like
435	2.85E-02	1.983	16708161	ENTPD7	ectonucleoside triphosphate diphosphohydrolase 7
436	4.56E-03	1.982	16732676	UBASH3B	ubiquitin associated and SH3 domain containing B
437	4.26E-02	1.980	16818630	PHKB	phosphorylase kinase regulatory subunit beta
438	3.60E-02	1.979	17096471	TBC1D2	TBC1 domain family member 2
439	1.59E-02	1.978	16721375	SMPD1	sphingomyelin phosphodiesterase 1
440	2.96E-02	1.976	16887360	UBR3	ubiquitin protein ligase E3 component n-recognin 3 (putative)
441	2.61E-02	1.976	17058878	TMEM120A	transmembrane protein 120A
442	3.93E-03	1.974	16936947	ITPR1	inositol 1,4,5-trisphosphate receptor type 1
443	1.29E-02	1.974	17017147	HLA-B	major histocompatibility complex, class I, B
444	4.01E-02	1.974	16971943	ETFDH	electron transfer flavoprotein dehydrogenase
445	2.28E-02	1.972	16798224	SNRPN	small nuclear ribonucleoprotein polypeptide N
446	2.34E-02	1.971	17104051	TRO	trophinin
447	1.72E-03	1.971	16836214	TOM1L1	target of myb1 like 1 membrane trafficking protein
448	2.09E-02	1.970	16896442	EIF2AK2	eukaryotic translation initiation factor 2 alpha kinase 2
449	4.32E-02	1.969	16712773	MKX	mohawk homeobox
450	3.40E-02	1.968	16889879	NRP2	neuropilin 2
451	1.28E-02	1.967	16906991	RFTN2	raftlin family member 2
452	2.41E-02	1.965	16855637	PIGN	phosphatidylinositol glycan anchor biosynthesis class N
453	3.58E-02	1.965	17084767	NPR2	natriuretic peptide receptor 2
454	7.55E-03	1.965	17108959	ANOS1	anosmin 1
455	6.31E-03	1.964	17072920	WISP1	WNT1 inducible signaling pathway protein 1
456	3.97E-02	1.963	17009620	TMEM14A	transmembrane protein 14A
457	3.36E-02	1.963	17020620	LMBRD1	LMBR1 domain containing 1
458	3.62E-02	1.961	16829037	ZCCHC14	zinc finger CCHC-type containing 14
459	1.32E-02	1.960	16693813	ADAR	adenosine deaminase, RNA specific
460	1.36E-02	1.959	17022943	TSPYL1	TSPY like 1
461	3.68E-02	1.959	16798220	SNRPN	small nuclear ribonucleoprotein polypeptide N
462	4.14E-02	1.959	16773552	ATP5EP2	ATP synthase, H+ transporting, mitochondrial F1 complex, epsilon subunit pseudogene 2

463	4.06E-02	1.958	16837128	PRKCA	protein kinase C alpha
464	6.50E-03	1.957	17013567	TAB2	TGF-beta activated kinase 1/MAP3K7 binding protein 2
465	2.74E-02	1.957	17009853	BAG2	BCL2 associated athanogene 2
466	2.66E-02	1.955	17055354	ETV1	ETS variant 1
467	4.06E-02	1.954	17052935	OR2A20P	olfactory receptor family 2 subfamily A member 20 pseudogene
468	2.35E-02	1.952	16990572	SH3RF2	SH3 domain containing ring finger 2
469	4.60E-02	1.950	16852666	SEC11C	SEC11 homolog C, signal peptidase complex subunit
470	3.01E-03	1.949	17009289	RUNX2	runt related transcription factor 2
471	3.23E-02	1.949	16813058	MFGE8	milk fat globule-EGF factor 8 protein
472	2.27E-03	1.947	16868371	ZNF266	zinc finger protein 266
473	4.00E-02	1.946	17058142	ZNF117	zinc finger protein 117
474	7.24E-03	1.946	17117888	ZEB2	zinc finger E-box binding homeobox 2
475	4.43E-03	1.943	16663033	SMAP2	small ArfGAP2
476	2.50E-02	1.943	16909828	COL6A3	collagen type VI alpha 3 chain
477	5.23E-04	1.942	16960114	PLOD2	procollagen-lysine,2-oxoglutarate 5-dioxygenase 2
478	4.40E-02	1.941	17045917	UPP1	uridine phosphorylase 1
479	4.79E-02	1.939	16848953	TRIM47	tripartite motif containing 47
480	4.06E-02	1.938	16956448	GBE1	glucan (1,4-alpha-), branching enzyme 1
481	1.06E-02	1.936	16980998	C4orf46	chromosome 4 open reading frame 46
482	7.80E-03	1.933	17048102	CFAP69	cilia and flagella associated protein 69
483	4.64E-02	1.931	16840318	NLRP1	NLR family pyrin domain containing 1
484	4.78E-03	1.931	16881802	DOK1	docking protein 1
485	3.40E-02	1.929	17016406	HIST1H4H	histone cluster 1, H4h
486	4.68E-02	1.929	17092153	AK3	adenylate kinase 3
487	1.98E-02	1.927	16808314	PPIP5K1	diphosphoinositol pentakisphosphate kinase 1
488	2.03E-02	1.923	16804942	FURIN	furin, paired basic amino acid cleaving enzyme
489	2.33E-02	1.922	16724471	DDB2	damage specific DNA binding protein 2
490	1.08E-03	1.920	17009862	PRIM2	primase (DNA) subunit 2
491	4.43E-02	1.918	16908415	ZNF142	zinc finger protein 142
492	3.55E-02	1.917	16942202	PXK	PX domain containing serine/threonine kinase like
493	4.14E-02	1.917	16826243	ITFG1	integrin alpha FG-GAP repeat containing 1
494	3.07E-02	1.917	16922243	IL10RB	interleukin 10 receptor subunit beta
495	4.16E-02	1.917	16749423	ARNTL2	aryl hydrocarbon receptor nuclear translocator like 2
496	1.72E-02	1.916	16735738	RNF141	ring finger protein 141
497	4.14E-02	1.916	16958303	CCDC14	coiled-coil domain containing 14
498	4.68E-02	1.915	16845518	TMEM101	transmembrane protein 101

499	2.66E-02	1.915	17088957	LOC100129034	uncharacterized LOC100129034
500	4.55E-02	1.914	16728066	LRP5	LDL receptor related protein 5
501	2.67E-02	1.913	17019549	MRPL14	mitochondrial ribosomal protein L14
502	4.42E-03	1.913	16984768	HSPB3	heat shock protein family B (small) member 3
503	3.41E-02	1.912	17092788	MIR4474	microRNA 4474
504	3.60E-02	1.910	16699932	TMEM63A	transmembrane protein 63A
505	5.06E-03	1.910	17092081	GLIS3	GLIS family zinc finger 3
506	3.38E-02	1.909	16874109	PLEKHA4	pleckstrin homology domain containing A4
507	2.23E-02	1.909	16707196	IFIT1	interferon induced protein with tetratricopeptide repeats 1
508	3.70E-02	1.907	16802106	HACD3	3-hydroxyacyl-CoA dehydratase 3
509	1.05E-02	1.907	16851749	DSG3	desmoglein 3
510	4.56E-02	1.906	16981157	TMEM192	transmembrane protein 192
511	3.48E-03	1.906	16664569	CDKN2C	cyclin dependent kinase inhibitor 2C
512	2.82E-02	1.905	16952659	POMGNT2	protein O-linked mannose N-acetylglucosaminyltransferase 2 (beta 1,4-)
513	1.42E-02	1.904	17017979	TAP1	transporter 1, ATP binding cassette subfamily B member
514	2.67E-02	1.904	16768738	NTN4	netrin 4
515	1.81E-02	1.901	16749459	PPFIBP1	PPFIA binding protein 1
516	2.11E-02	1.901	16990294	PCDHB10	protocadherin beta 10
517	1.29E-02	1.900	17095423	GOLM1	golgi membrane protein 1
518	1.89E-02	1.899	16811500	NPTN	neuroplastin
519	2.09E-02	1.899	17020317	DST	dystonin
520	3.36E-02	1.898	16835781	XYLT2	xylosyltransferase 2
521	4.79E-02	1.898	16757427	TPCN1	two pore segment channel 1
522	3.77E-02	1.898	16943954	SLC35A5	solute carrier family 35 member A5
523	3.86E-02	1.898	17114829	IDS	iduronate 2-sulfatase
524	3.12E-02	1.897	16966685	SPATA18	spermatogenesis associated 18
525	1.24E-02	1.897	16804490	ISG20	interferon stimulated exonuclease gene 20
526	3.06E-02	1.897	16694506	GLMP	glycosylated lysosomal membrane protein
527	5.16E-03	1.896	16767751	PHLDA1	pleckstrin homology like domain family A member 1
528	1.71E-02	1.896	16707202	IFIT5	interferon induced protein with tetratricopeptide repeats 5
529	1.45E-02	1.896	16852025	FHOD3	formin homology 2 domain containing 3
530	2.93E-02	1.895	16785938	TTC9	tetratricopeptide repeat domain 9
531	4.33E-02	1.894	17076415	FGFR1	fibroblast growth factor receptor 1
532	2.53E-02	1.893	16953424	PFKFB4	6-phosphofructo-2-kinase/fructose-2,6-biphosphatase 4
533	1.53E-02	1.892	17075082	ASAH1	N-acylsphingosine amidohydrolase 1
534	4.74E-03	1.891	16662584	SH3D21	SH3 domain containing 21

535	1.26E-04	1.891	17005197	RNF144B	ring finger protein 144B
536	4.44E-02	1.891	16965642	RBPJ	recombination signal binding protein for immunoglobulin kappa J region
537	1.17E-02	1.890	17054149	WDR60	WD repeat domain 60
538	1.74E-02	1.889	16965719	STIM2	stromal interaction molecule 2
539	3.57E-02	1.889	17006142	HLA-A	major histocompatibility complex, class I, A
540	2.22E-02	1.889	17018788	BTBD9	BTB domain containing 9
541	3.93E-02	1.888	16828841	ZDHHC7	zinc finger DHHC-type containing 7
542	4.49E-02	1.888	16950632	EMC3	ER membrane protein complex subunit 3
543	8.80E-03	1.886	16701841	PFKP	phosphofructokinase, platelet
544	2.67E-02	1.885	16695258	PIGM	phosphatidylinositol glycan anchor biosynthesis class M
545	3.92E-02	1.885	17066523	BMP1	bone morphogenetic protein 1
546	2.31E-03	1.883	16908338	TMBIM1	transmembrane BAX inhibitor motif containing 1
547	3.03E-03	1.882	16957636	ZBTB20	zinc finger and BTB domain containing 20
548	1.14E-02	1.881	16827833	MTSS1L	metastasis suppressor 1 like
549	4.93E-02	1.880	17111599	LINC01278	long intergenic non-protein coding RNA 1278
550	3.34E-02	1.878	16870746	ZNF506	zinc finger protein 506
551	4.14E-03	1.878	16748888	PLEKHA5	pleckstrin homology domain containing A5
552	4.51E-02	1.878	17079517	LOC105375666	
553	4.62E-02	1.878	17058223	GS1-124K5.11	RAB guanine nucleotide exchange factor 1 pseudogene
554	2.07E-02	1.877	16950848	RAF1	Raf-1 proto-oncogene, serine/threonine kinase
555	2.85E-02	1.877	16990288	PCDHB16	protocadherin beta 16
556	4.30E-02	1.876	16875014	ZNF841	zinc finger protein 841
557	3.24E-02	1.876	16765325	MAP3K12	mitogen-activated protein kinase kinase kinase 12
558	4.50E-02	1.875	17015919	KIF13A	kinesin family member 13A
559	4.41E-02	1.875	16662890	BMP8A	bone morphogenetic protein 8a
560	3.09E-02	1.874	16966026	FAM114A1	family with sequence similarity 114 member A1
561	4.86E-02	1.872	16679003	GPR137B	G protein-coupled receptor 137B
562	1.91E-02	1.871	16960888	SHOX2	short stature homeobox 2
563	4.62E-02	1.870	16855138	DYM	dymeclin
564	3.67E-02	1.870	16978959	CASP6	caspase 6
565	4.08E-02	1.869	16774188	COG6	component of oligomeric golgi complex 6
566	3.94E-02	1.868	17113744	CUL4B	cullin 4B
567	6.60E-04	1.865	16855781	DSEL	dermatan sulfate epimerase-like
568	4.19E-02	1.863	17097731	PAPPA-AS1	PAPPA antisense RNA 1
569	1.16E-03	1.863	17110289	MAOB	monoamine oxidase B
570	3.50E-02	1.863	16879721	EPAS1	endothelial PAS domain protein 1

571	3.29E-02	1.862	16960084	SLC9A9	solute carrier family 9 member A9
572	5.85E-03	1.862	16879500	PLEKHH2	pleckstrin homology, MyTH4 and FERM domain containing H2
573	2.60E-02	1.862	16692667	MTMR11	myotubularin related protein 11
574	3.81E-02	1.862	16768413	DCN	decorin
575	2.07E-02	1.861	16974830	PPARGC1A	PPARG coactivator 1 alpha
576	4.83E-02	1.856	16904410	FIGN	fidgetin, microtubule severing factor
577	2.81E-02	1.854	16681408	SLC2A5	solute carrier family 2 member 5
578	2.46E-02	1.854	17024079	MAP3K5	mitogen-activated protein kinase kinase kinase 5
579	2.03E-02	1.854	16776961	GAS6-AS2	GAS6 antisense RNA 2 (head to head)
580	3.08E-02	1.853	16866131	ZNF776	zinc finger protein 776
581	2.15E-02	1.853	16838207	SEPT9	septin 9
582	1.32E-02	1.852	16920047	PREX1	phosphatidylinositol-3,4,5-trisphosphate dependent Rac exchange factor 1
583	4.38E-02	1.852	16748529	GPRC5A	G protein-coupled receptor class C group 5 member A
584	1.80E-02	1.852	16971806	GLRB	glycine receptor beta
585	7.15E-03	1.852	16944654	FAM162A	family with sequence similarity 162 member A
586	4.62E-02	1.852	16777198	CRYL1	crystallin lambda 1
587	1.85E-02	1.851	16836232	STXBP4	syntaxin binding protein 4
588	1.61E-02	1.851	16907526	INO80D	INO80 complex subunit D
589	6.98E-03	1.849	16823825	EMP2	epithelial membrane protein 2
590	1.91E-02	1.848	17044253	GPNMB	glycoprotein nmb
591	4.96E-02	1.847	16669721	NBPF15 (includes others)	neuroblastoma breakpoint family member 15
592	2.13E-02	1.846	16996298	IL6ST	interleukin 6 signal transducer
593	1.78E-02	1.846	16773759	FRY	FRY microtubule binding protein
594	9.42E-03	1.846	16762941	DENND5B	DENN domain containing 5B
595	1.47E-02	1.844	16728189	TPCN2	two pore segment channel 2
596	3.12E-02	1.844	17009659	FBXO9	F-box protein 9
597	4.76E-02	1.843	16753064	ARHGEF25	Rho guanine nucleotide exchange factor 25
598	1.26E-02	1.842	16819207	MT2A	metallothionein 2A
599	4.11E-02	1.842	16714060	ARHGAP22-IT1	ARHGAP22 intronic transcript 1
600	4.32E-02	1.841	16816140	PDXDC1	pyridoxal dependent decarboxylase domain containing 1
601	1.93E-03	1.840	16689020	MCOLN3	mucolipin 3
602	4.09E-02	1.839	17010715	CYB5R4	cytochrome b5 reductase 4
603	3.20E-02	1.838	16870522	SUGP2	SURP and G-patch domain containing 2
604	3.48E-02	1.838	17063975	CTAGE9 (includes others)	CTAGE family member 4
605	9.70E-03	1.837	16925057	SYNJ1	synaptojanin 1
606	3.62E-02	1.837	16709128	DUSP5	dual specificity phosphatase 5

607	3.56E-02	1.837	16915245	APCDD1L-AS1	APCDD1L antisense RNA 1 (head to head)
608	2.57E-02	1.836	16934140	PRR14L	proline rich 14 like
609	1.98E-03	1.835	16773165	TNFRSF19	TNF receptor superfamily member 19
610	2.46E-03	1.835	16844775	KRT19	keratin 19
611	2.97E-02	1.833	16979432	NDNF	neuron derived neurotrophic factor
612	4.16E-02	1.833	16867240	MAP2K2	mitogen-activated protein kinase kinase 2
613	3.43E-02	1.833	16864275	CPT1C	carnitine palmitoyltransferase 1C
614	2.16E-02	1.832	16691893	LINC00869	long intergenic non-protein coding RNA 869
615	4.09E-02	1.831	16941088	MAPKAPK3	mitogen-activated protein kinase-activated protein kinase 3
616	2.91E-02	1.830	16686060	SLC2A1	solute carrier family 2 member 1
617	1.14E-02	1.830	16733985	MIR210HG	MIR210 host gene
618	8.97E-03	1.830	16982607	LOC100288152	uncharacterized LOC100288152
619	9.66E-03	1.829	16982269	FAT1	FAT atypical cadherin 1
620	2.09E-02	1.829	16840513	DLG4	discs large MAGUK scaffold protein 4
621	1.60E-02	1.829	16671579	ADAM15	ADAM metallopeptidase domain 15
622	3.03E-02	1.826	16780808	KDELC1	KDEL motif containing 1
623	9.67E-03	1.826	16938271	KAT2B	lysine acetyltransferase 2B
624	3.82E-02	1.826	16747184	CD9	CD9 molecule
625	2.43E-02	1.825	17091883	FAM157A/FAM157B	family with sequence similarity 157 member A
626	6.62E-03	1.824	16953753	SLC25A20	solute carrier family 25 member 20
627	1.69E-02	1.824	16989897	CXXC5	CXXC finger protein 5
628	2.04E-03	1.823	16778355	LHFP	lipoma HMGIC fusion partner
629	2.99E-02	1.822	16771652	VPS33A	VPS33A, CORVET/HOPS core subunit
630	4.88E-02	1.822	16747257	LOC105369628	uncharacterized LOC105369628
631	1.99E-02	1.821	16753630	LOC105369807	uncharacterized LOC105369807
632	1.40E-03	1.821	16889411	CFLAR	CASP8 and FADD like apoptosis regulator
633	3.21E-02	1.820	16757225	TRAFD1	TRAF-type zinc finger domain containing 1
634	3.27E-02	1.820	16850865	ANKRD12	ankyrin repeat domain 12
635	2.21E-02	1.818	16936397	MAPK12	mitogen-activated protein kinase 12
636	2.38E-02	1.818	16897719	CCDC88A	coiled-coil domain containing 88A
637	2.80E-02	1.818	17090373	ABL1	ABL proto-oncogene 1, non-receptor tyrosine kinase
638	1.78E-02	1.816	16909165	IRS1	insulin receptor substrate 1
639	3.41E-02	1.816	16794220	DCAF5	DDB1 and CUL4 associated factor 5
640	5.51E-03	1.815	16719562	EBF3	early B-cell factor 3
641	1.33E-02	1.815	16851866	DTNA	dystrobrevin alpha
642	1.49E-02	1.812	17092767	MLLT3	MLLT3, super elongation complex subunit

643	4.09E-02	1.812	16924305	BTG3	BTG anti-proliferation factor 3
644	1.97E-02	1.810	16667498	SNX7	sorting nexin 7
645	1.12E-02	1.809	16679764	OR2L8	olfactory receptor family 2 subfamily L member 8 (gene/pseudogene)
646	3.68E-03	1.809	16815791	ATF7IP2	activating transcription factor 7 interacting protein 2
647	2.47E-02	1.807	16951247	ANKRD28	ankyrin repeat domain 28
648	1.14E-02	1.806	16744289	PPP2R1B	protein phosphatase 2 scaffold subunit Abeta
649	4.46E-02	1.805	16965338	MED28	mediator complex subunit 28
650	7.06E-03	1.805	16690139	EXTL2	exostosin like glycosyltransferase 2
651	4.46E-02	1.804	16706135	ZSWIM8	zinc finger SWIM-type containing 8
652	1.99E-02	1.804	16838581	RNF213	ring finger protein 213
653	3.46E-02	1.804	16794705	NPC2	NPC intracellular cholesterol transporter 2
654	4.60E-02	1.804	16811469	ADPGK	ADP dependent glucokinase
655	4.94E-02	1.803	17086596	SPIN1	spindlin 1
656	7.25E-03	1.803	16801283	PIGB	phosphatidylinositol glycan anchor biosynthesis class B
657	1.98E-02	1.802	16841982	TOM1L2	target of myb1 like 2 membrane trafficking protein
658	1.05E-03	1.802	16791393	CBLN3	cerebellin 3 precursor
659	4.37E-02	1.802	16835816	ACSF2	acyl-CoA synthetase family member 2
660	1.23E-02	1.801	17055763	STEAP1B	STEAP family member 1B
661	4.85E-02	1.800	16826639	CES1	carboxylesterase 1
662	2.12E-02	1.799	16691668	NOTCH2	notch 2
663	1.44E-02	1.799	17023927	AHI1	Abelson helper integration site 1
664	2.72E-03	1.798	16858007	ZNF561-AS1	ZNF561 antisense RNA 1 (head to head)
665	3.66E-02	1.797	17063981	TCAF1	TRPM8 channel associated factor 1
666	1.57E-02	1.797	17019820	PTCHD4	patched domain containing 4
667	4.36E-02	1.796	17021004	PHIP	pleckstrin homology domain interacting protein
668	2.43E-02	1.796	16901393	FHL2	four and a half LIM domains 2
669	8.13E-03	1.795	16892521	SCARNA5	small Cajal body-specific RNA 5
670	4.64E-02	1.794	17075529	ENTPD4	ectonucleoside triphosphate diphosphohydrolase 4
671	2.00E-02	1.794	17054823	ACTB	actin beta
672	2.22E-02	1.793	16798244	SNRPN	small nuclear ribonucleoprotein polypeptide N
673	6.76E-03	1.792	16931315	PRR34-AS1	PRR34 antisense RNA 1
674	3.40E-02	1.792	17060758	EPHB4	EPH receptor B4
675	4.78E-02	1.791	16907257	TRAK2	trafficking kinesin protein 2
676	1.37E-02	1.790	17063536	SLC37A3	solute carrier family 37 member 3
677	2.86E-02	1.788	17082962	SPIDR	scaffolding protein involved in DNA repair
678	1.91E-02	1.788	16665182	PRKAA2	protein kinase AMP-activated catalytic subunit alpha 2

679	3.72E-02	1.788	16922759	KCNJ15	potassium voltage-gated channel subfamily J member 15
680	4.17E-02	1.788	16851249	ANKRD20A5P	ankyrin repeat domain 20 family member A5, pseudogene
681	4.58E-02	1.787	17071321	VPS13B	vacuolar protein sorting 13 homolog B
682	3.32E-02	1.786	16843585	TBC1D3 (includes others)	TBC1 domain family member 3
683	1.54E-02	1.785	16987673	PAM	peptidylglycine alpha-amidating monooxygenase
684	3.26E-02	1.784	16866016	TRAPPC2B	trafficking protein particle complex 2B
685	3.39E-02	1.784	17045542	STK17A	serine/threonine kinase 17a
686	5.23E-03	1.784	16710720	DPYSL4	dihydropyrimidinase like 4
687	3.89E-02	1.782	16997212	GFM2	G elongation factor mitochondrial 2
688	3.82E-02	1.781	16981516	LOC105377540	uncharacterized LOC105377540
689	9.90E-03	1.781	17006261	HLA-E	major histocompatibility complex, class I, E
690	2.66E-02	1.780	16834533	RUNDC1	RUN domain containing 1
691	5.06E-04	1.780	16749504	REP15	RAB15 effector protein
692	3.30E-02	1.780	16726081	RARRES3	retinoic acid receptor responder 3
693	4.82E-02	1.780	16825333	CLN3	CLN3, battenin
694	4.00E-02	1.779	16756895	IFT81	intraflagellar transport 81
695	2.23E-02	1.779	16747481	GNB3	G protein subunit beta 3
696	3.29E-02	1.778	16872203	PAF1	PAF1 homolog, Paf1/RNA polymerase II complex component
697	2.81E-02	1.777	16832835	SUZ12P1	SUZ12 polycomb repressive complex 2 subunit pseudogene 1
698	4.99E-02	1.777	17101292	STS	steroid sulfatase (microsomal), isozyme S
699	2.49E-02	1.775	16784071	TMX1	thioredoxin related transmembrane protein 1
700	2.42E-02	1.775	16927040	MRPL40	mitochondrial ribosomal protein L40
701	1.67E-02	1.774	16881434	SPR	sepiapterin reductase (7,8-dihydrobiopterin:NADP+ oxidoreductase)
702	3.96E-02	1.773	17058731	GTF2IP1	general transcription factor IIi pseudogene 1
703	4.14E-03	1.772	16671457	IL6R	interleukin 6 receptor
704	3.66E-04	1.771	17097000	PTPN3	protein tyrosine phosphatase, non-receptor type 3
705	3.96E-02	1.771	17046886	GTF2IP4	general transcription factor IIi pseudogene 4
706	1.71E-02	1.770	16994272	SEMA5A	semaphorin 5A
707	2.22E-02	1.770	16983843	LOC646652	integral membrane glycoprotein-like
708	1.83E-02	1.770	16896576	LOC102723739	uncharacterized LOC102723739
709	1.28E-02	1.769	16866153	ZNF417/ZNF587	zinc finger protein 587
710	3.64E-02	1.769	16669983	LOC101060254	myomegalin-like
711	2.13E-03	1.769	16785789	GALNT16	polypeptide N-acetylgalactosaminyltransferase 16
712	3.89E-02	1.768	16850896	TWSG1	twisted gastrulation BMP signaling modulator 1
713	9.48E-03	1.768	16946393	RASA2	RAS p21 protein activator 2
714	2.42E-02	1.767	16684581	SPOCD1	SPOC domain containing 1

715	3.36E-02	1.767	16996089	MOCS2	molybdenum cofactor synthesis 2
716	2.53E-02	1.766	17114707	LDOC1	leucine zipper down-regulated in cancer 1
717	9.01E-03	1.765	16935905	KIAA1644	KIAA1644
718	2.47E-02	1.762	16800229	MAP1A	microtubule associated protein 1A
719	7.24E-03	1.761	16869324	PRDX2	peroxiredoxin 2
720	3.85E-02	1.761	17112269	ATRX	ATRX, chromatin remodeler
721	4.07E-02	1.760	17068541	VDAC3	voltage dependent anion channel 3
722	4.54E-02	1.759	16859763	IFI30	IFI30, lysosomal thiol reductase
723	2.31E-02	1.758	16663871	DMAP1	DNA methyltransferase 1 associated protein 1
724	2.55E-02	1.757	17063428	ZC3HAV1	zinc finger CCCH-type containing, antiviral 1
725	6.38E-03	1.756	16901427	UXS1	UDP-glucuronate decarboxylase 1
726	3.53E-02	1.755	16897112	PREPL	prolyl endopeptidase-like
727	1.31E-02	1.755	16800980	GABPB1-AS1	GABPB1 antisense RNA 1
728	1.33E-02	1.754	17111435	WNK3	WNK lysine deficient protein kinase 3
729	3.57E-02	1.754	16854904	PSTPIP2	proline-serine-threonine phosphatase interacting protein 2
730	5.53E-03	1.754	16706180	PLAU	plasminogen activator, urokinase
731	4.35E-02	1.753	16715529	USP54	ubiquitin specific peptidase 54
732	9.36E-03	1.753	16928428	GRK3	G protein-coupled receptor kinase 3
733	1.28E-02	1.752	17075829	ZNF395	zinc finger protein 395
734	4.19E-02	1.752	17045354	YAE1D1	Yae1 domain containing 1
735	3.50E-02	1.752	16808940	SECISBP2L	SECIS binding protein 2 like
736	3.23E-02	1.751	16709964	WDR11	WD repeat domain 11
737	4.64E-02	1.751	16889762	CYP20A1	cytochrome P450 family 20 subfamily A member 1
738	4.70E-02	1.751	17111611	ARHGEF9	Cdc42 guanine nucleotide exchange factor 9
739	3.08E-02	1.751	16900628	ANKRD39	ankyrin repeat domain 39
740	3.11E-02	1.750	16666471	PTGFR	prostaglandin F receptor
741	1.99E-02	1.750	16958251	MYLK	myosin light chain kinase
742	2.66E-02	1.750	16989367	DDX46	DEAD-box helicase 46
743	6.08E-03	1.749	16991151	SYNPO	synaptopodin
744	1.77E-02	1.749	16978969	PLA2G12A	phospholipase A2 group XIIA
745	3.92E-02	1.749	16874313	PIH1D1	PIH1 domain containing 1
746	3.73E-02	1.747	17009093	VEGFA	vascular endothelial growth factor A
747	2.09E-02	1.747	16991180	SMIM3	small integral membrane protein 3
748	2.61E-02	1.746	16821047	ZNRF1	zinc and ring finger 1, E3 ubiquitin protein ligase
749	4.85E-02	1.745	16973771	MFSD10	major facilitator superfamily domain containing 10
750	1.15E-02	1.745	16695268	IGSF8	immunoglobulin superfamily member 8

751	4.06E-02	1.745	16692892	CERS2	ceramide synthase 2
752	2.46E-02	1.744	16777651	LNX2	ligand of numb-protein X 2
753	2.90E-02	1.743	16959925	XRN1	5'-3' exoribonuclease 1
754	2.75E-02	1.743	16796854	MOK	MOK protein kinase
755	2.30E-02	1.743	16670332	LINC00869	long intergenic non-protein coding RNA 869
756	7.17E-03	1.743	16968765	HERC5	HECT and RLD domain containing E3 ubiquitin protein ligase 5
757	2.02E-02	1.743	16855358	CCDC68	coiled-coil domain containing 68
758	4.25E-02	1.742	16687273	ECHDC2	enoyl-CoA hydratase domain containing 2
759	4.74E-02	1.741	16886331	MBD5	methyl-CpG binding domain protein 5
760	4.56E-02	1.741	17117744	LOC100507131	uncharacterized LOC100507131
761	1.14E-02	1.741	17016762	HCG4	HLA complex group 4 (non-protein coding)
762	1.71E-02	1.740	16722684	ZDHHC13	zinc finger DHHC-type containing 13
763	1.74E-04	1.740	16909257	SLC19A3	solute carrier family 19 member 3
764	4.10E-02	1.740	16672279	KIRREL	kin of IRRE like (Drosophila)
765	2.24E-02	1.739	17005904	ZKSCAN8	zinc finger with KRAB and SCAN domains 8
766	3.52E-02	1.739	16922078	KRTAP20-1	keratin associated protein 20-1
767	8.35E-03	1.739	16718622	ABLIM1	actin binding LIM protein 1
768	4.91E-02	1.737	17053892	INSIG1	insulin induced gene 1
769	8.88E-03	1.737	16955535	ACOX2	acyl-CoA oxidase 2
770	4.55E-02	1.736	17108067	SLC6A8	solute carrier family 6 member 8
771	3.26E-02	1.734	16951990	SUSD5	sushi domain containing 5
772	8.25E-03	1.734	17103837	MAGED4/MAGED4B	MAGE family member D4B
773	4.02E-02	1.733	16811372	PKM	pyruvate kinase, muscle
774	6.10E-03	1.733	17111025	MAGED4/MAGED4B	MAGE family member D4B
775	2.03E-02	1.732	16808316	PPIP5K1	diphosphoinositol pentakisphosphate kinase 1
776	3.96E-02	1.732	17108428	PLXNA3	plexin A3
777	2.29E-02	1.731	16698136	CYB5R1	cytochrome b5 reductase 1
778	3.52E-02	1.730	16988376	HSD17B4	hydroxysteroid 17-beta dehydrogenase 4
779	4.25E-02	1.728	16798196	SNRPN	small nuclear ribonucleoprotein polypeptide N
780	3.05E-02	1.728	16995140	RAD1	RAD1 checkpoint DNA exonuclease
781	4.01E-02	1.728	17105515	ARMCX1	armadillo repeat containing, X-linked 1
782	3.11E-02	1.727	17023677	STX7	syntaxin 7
783	2.79E-02	1.727	16813871	CHSY1	chondroitin sulfate synthase 1
784	2.15E-02	1.727	16730471	CEP126	centrosomal protein 126
785	4.95E-02	1.726	16926942	USP18	ubiquitin specific peptidase 18
786	2.45E-03	1.726	16753641	HMGA2	high mobility group AT-hook 2

787	1.14E-02	1.725	16939558	CTNNB1	catenin beta 1
788	2.07E-02	1.724	16862262	SHKBP1	SH3KBP1 binding protein 1
789	3.78E-02	1.724	16691929	LINC00623	long intergenic non-protein coding RNA 623
790	4.58E-02	1.723	16665656	RAVER2	ribonucleoprotein, PTB binding 2
791	3.07E-02	1.723	16997503	LHFPL2	lipoma HMGIC fusion partner-like 2
792	4.76E-02	1.722	16905521	TTC30A	tetratricopeptide repeat domain 30A
793	4.19E-02	1.722	16706990	MINPP1	multiple inositol-polyphosphate phosphatase 1
794	9.43E-05	1.722	16689352	GBP2	guanylate binding protein 2
795	4.51E-02	1.722	16725049	FAM111A	family with sequence similarity 111 member A
796	3.02E-02	1.721	17045303	STARD3NL	STARD3 N-terminal like
797	1.88E-02	1.721	17017126	HLA-C	major histocompatibility complex, class I, C
798	3.22E-02	1.720	16989380	TXNDC15	thioredoxin domain containing 15
799	2.01E-02	1.720	16851565	TTC39C	tetratricopeptide repeat domain 39C
800	3.36E-02	1.720	16766281	SNORD59A	small nucleolar RNA, C/D box 59A
801	2.42E-02	1.720	16849913	ARL16	ADP ribosylation factor like GTPase 16
802	1.93E-02	1.719	16990312	PCDHB13	protocadherin beta 13
803	5.29E-03	1.716	16930199	APOBEC3G	apolipoprotein B mRNA editing enzyme catalytic subunit 3G
804	2.65E-02	1.715	16784333	SAMD4A	sterile alpha motif domain containing 4A
805	4.39E-02	1.714	16873060	PLAUR	plasminogen activator, urokinase receptor
806	4.78E-02	1.714	16864234	BCL2L12	BCL2 like 12
807	8.90E-03	1.712	16744731	SIK3	SIK family kinase 3
808	4.14E-02	1.712	16667530	PALMD	palmdelphin
809	1.56E-02	1.711	16709537	TRUB1	TruB pseudouridine synthase family member 1
810	3.89E-03	1.711	16832168	SPECC1	sperm antigen with calponin homology and coiled-coil domains 1
811	3.33E-02	1.711	16968077	CCNG2	cyclin G2
812	7.70E-03	1.710	16928988	TCN2	transcobalamin 2
813	3.38E-02	1.710	16808326	PPIP5K1	diphosphoinositol pentakisphosphate kinase 1
814	1.54E-02	1.710	17088929	NEK6	NIMA related kinase 6
815	3.09E-02	1.710	16866974	GNG7	G protein subunit gamma 7
816	8.76E-03	1.709	16956285	PDZRN3	PDZ domain containing ring finger 3
817	3.69E-02	1.708	17068724	SPIDR	scaffolding protein involved in DNA repair
818	4.92E-02	1.708	16808304	PPIP5K1	diphosphoinositol pentakisphosphate kinase 1
819	2.21E-02	1.708	16724701	LOC105369150	uncharacterized LOC105369150
820	3.44E-02	1.708	16740770	B4GAT1	beta-1,4-glucuronyltransferase 1
821	3.58E-02	1.708	17077760	ARMC1	armadillo repeat containing 1
822	4.17E-02	1.707	17077848	VCPIP1	valosin containing protein interacting protein 1

823	6.15E-03	1.707	16673748	FMO4	flavin containing monooxygenase 4
824	1.98E-02	1.707	16942681	EBLN2	endogenous Bornavirus-like nucleoprotein 2
825	4.03E-02	1.707	16820345	DUS2	dihydrouridine synthase 2
826	1.97E-02	1.707	16953778	DALRD3	DALR anticodon binding domain containing 3
827	3.98E-02	1.707	16657598	AGRN	agrin
828	1.91E-02	1.706	16926460	ITGB2	integrin subunit beta 2
829	3.75E-02	1.705	16874273	TEAD2	TEA domain transcription factor 2
830	7.33E-04	1.705	16798238	SNRPN	small nuclear ribonucleoprotein polypeptide N
831	9.56E-03	1.704	16929064	SMTN	smoothelin
832	4.25E-02	1.704	16854727	SLC39A6	solute carrier family 39 member 6
833	2.92E-02	1.704	17086540	CTSL	cathepsin L
834	1.70E-02	1.702	16823666	PPL	periplakin
835	3.12E-02	1.702	16714200	PARG	poly(ADP-ribose) glycohydrolase
836	3.21E-02	1.701	16787814	IFI27	interferon alpha inducible protein 27
837	3.38E-02	1.701	17101422	CLCN4	chloride voltage-gated channel 4
838	3.28E-02	1.700	16836697	BCAS3	BCAS3, microtubule associated cell migration factor
839	2.90E-02	1.698	16770344	HECTD4	HECT domain E3 ubiquitin protein ligase 4
840	2.91E-02	1.697	17046547	ZNF92	zinc finger protein 92
841	9.67E-03	1.697	17073259	LY6E	lymphocyte antigen 6 complex, locus E
842	1.96E-03	1.697	16730429	ARHGAP42	Rho GTPase activating protein 42
843	3.30E-02	1.696	16689664	TMED5	transmembrane p24 trafficking protein 5
844	4.27E-02	1.696	16788695	SNORD114-27	small nucleolar RNA, C/D box 114-27
845	4.43E-02	1.696	16796207	DDX24	DEAD-box helicase 24
846	3.07E-02	1.696	16813286	CIB1	calcium and integrin binding 1
847	2.18E-02	1.695	16758817	ZNF664	zinc finger protein 664
848	2.89E-02	1.695	16751083	LARP4	La ribonucleoprotein domain family member 4
849	1.45E-02	1.695	17045938	ABCA13	ATP binding cassette subfamily A member 13
850	5.03E-03	1.694	16863344	QPCTL	glutaminyl-peptide cyclotransferase like
851	2.09E-02	1.694	16931333	MIRLET7BHG	MIRLET7B host gene
852	4.20E-02	1.694	16891176	ANKZF1	ankyrin repeat and zinc finger domain containing 1
853	2.40E-02	1.693	17012379	CENPW	centromere protein W
854	4.04E-02	1.692	16685099	KIAA0319L	KIAA0319 like
855	7.00E-03	1.692	16705116	FAM133CP	family with sequence similarity 133, member A pseudogene
856	2.17E-02	1.691	17103303	PORCN	porcupine homolog (Drosophila)
857	4.24E-02	1.691	16934109	PISD	phosphatidylserine decarboxylase
858	3.36E-02	1.691	16851353	MIB1	mindbomb E3 ubiquitin protein ligase 1

859	4.56E-02	1.691	16777185	GJB2	gap junction protein beta 2
860	2.00E-02	1.690	17053906	LOC100286906	uncharacterized LOC100286906
861	3.63E-02	1.690	17095098	GNA14	G protein subunit alpha 14
862	3.35E-02	1.689	16775520	CLN5	ceroid-lipofuscinosis, neuronal 5
863	7.21E-03	1.689	17105655	BHLHB9	basic helix-loop-helix domain containing, class B, 9
864	3.75E-02	1.689	16924153	BAGE2	B melanoma antigen family member 2
865	4.52E-02	1.688	16767052	GNS	glucosamine (N-acetyl)-6-sulfatase
866	3.63E-02	1.688	16899686	GGCX	gamma-glutamyl carboxylase
867	4.17E-02	1.688	17098506	FAM129B	family with sequence similarity 129 member B
868	4.29E-02	1.688	16992557	CPEB4	cytoplasmic polyadenylation element binding protein 4
869	3.07E-02	1.687	16884441	ZC3H6	zinc finger CCCH-type containing 6
870	2.37E-02	1.685	17074029	TDRP	testis development related protein
871	3.66E-02	1.685	16863884	NUCB1	nucleobindin 1
872	2.05E-02	1.685	16668817	MOV10	Mov10 RISC complex RNA helicase
873	1.74E-02	1.684	17018215	TAPBP	TAP binding protein (tapasin)
874	2.58E-02	1.683	16864737	mir-10	microRNA 100
875	4.19E-02	1.683	16980918	CTSO	cathepsin O
876	1.74E-02	1.683	16944010	BOC	BOC cell adhesion associated, oncogene regulated
877	3.69E-02	1.682	16663792	ATP6V0B	ATPase H+ transporting V0 subunit b
878	2.09E-02	1.681	17087107	C9orf3	chromosome 9 open reading frame 3
879	2.87E-02	1.680	16665757	PDE4B	phosphodiesterase 4B
880	4.93E-02	1.680	16684036	AHDC1	AT-hook DNA binding motif containing 1
881	3.63E-02	1.679	16798234	SNRPN	small nuclear ribonucleoprotein polypeptide N
882	4.15E-02	1.679	16879276	MORN2	MORN repeat containing 2
883	3.32E-02	1.678	16843608	TBC1D3 (includes others)	TBC1 domain family member 3
884	4.38E-02	1.677	16933044	ZNF70	zinc finger protein 70
885	4.70E-02	1.677	16682485	mir-1290	microRNA 1290
886	4.16E-02	1.677	16835071	LRRC37A3 (includes others)	leucine rich repeat containing 37 member A3
887	2.70E-02	1.677	16670114	BCL9	B-cell CLL/lymphoma 9
888	1.27E-02	1.676	16975777	TEC	tec protein tyrosine kinase
889	4.33E-02	1.676	16906830	C2orf66	chromosome 2 open reading frame 66
890	4.21E-02	1.674	16735846	ZBED5	zinc finger BED-type containing 5
891	2.34E-02	1.674	17105010	COX7B	cytochrome c oxidase subunit 7B
892	1.81E-02	1.674	16739483	BSCL2	BSCL2, seipin lipid droplet biogenesis associated
893	1.79E-02	1.673	16722667	TMEM86A	transmembrane protein 86A

894	2.09E-02	1.673	16670774	PIP5K1A	phosphatidylinositol-4-phosphate 5-kinase type 1 alpha
895	3.37E-02	1.671	16716870	TCTN3	tectonic family member 3
896	1.56E-02	1.671	17117726	CNP	2',3'-cyclic nucleotide 3' phosphodiesterase
897	6.53E-03	1.670	17006122	HLA-G	major histocompatibility complex, class I, G
898	1.72E-02	1.670	16956983	CBLB	Cbl proto-oncogene B
899	4.52E-02	1.669	16699458	RAB3GAP2	RAB3 GTPase activating non-catalytic protein subunit 2
900	4.47E-02	1.669	16929849	PDXP	pyridoxal phosphatase
901	1.84E-02	1.669	17049814	ORAI2	ORAI calcium release-activated calcium modulator 2
902	6.33E-03	1.669	16977925	FAM13A	family with sequence similarity 13 member A
903	4.35E-02	1.668	16838271	TNRC6C	trinucleotide repeat containing 6C
904	3.71E-02	1.668	16835213	NPEPPS	aminopeptidase puromycin sensitive
905	1.90E-02	1.668	16670739	MLLT11	myeloid/lymphoid or mixed-lineage leukemia; translocated to, 11
906	2.21E-02	1.668	16762978	AMN1	antagonist of mitotic exit network 1 homolog
907	2.76E-02	1.667	16709072	ADD3	adducin 3
908	5.46E-03	1.665	16782355	THTPA	thiamine triphosphatase
909	1.41E-02	1.665	16734371	IGF2	insulin like growth factor 2
910	3.73E-02	1.665	17022553	DDO	D-aspartate oxidase
911	2.40E-02	1.664	17071208	LAPTM4B	lysosomal protein transmembrane 4 beta
912	7.65E-03	1.664	16809511	FAM214A	family with sequence similarity 214 member A
913	3.31E-02	1.663	16826966	SNORA46	small nucleolar RNA, H/ACA box 46
914	2.01E-03	1.663	16944798	KALRN	kalirin, RhoGEF kinase
915	9.56E-03	1.662	16658064	TNFRSF14	TNF receptor superfamily member 14
916	3.44E-02	1.662	16818204	KAT8	lysine acetyltransferase 8
917	3.94E-02	1.661	17107717	MTMR1	myotubularin related protein 1
918	2.67E-02	1.661	17051167	METTL2B	methyltransferase like 2B
919	3.26E-02	1.660	16788681	SNORD114-20	small nucleolar RNA, C/D box 114-20
920	3.56E-02	1.660	16850232	OGFOD3	2-oxoglutarate and iron dependent oxygenase domain containing 3
921	3.01E-02	1.660	17084904	MELK	maternal embryonic leucine zipper kinase
922	1.61E-02	1.659	16898728	TIA1	TIA1 cytotoxic granule-associated RNA binding protein
923	1.05E-02	1.659	16940211	TDGF1	teratocarcinoma-derived growth factor 1
924	4.87E-02	1.659	17010809	SMIM8	small integral membrane protein 8
925	8.78E-03	1.659	16835064	NSF	N-ethylmaleimide sensitive factor, vesicle fusing ATPase
926	8.78E-03	1.659	16850337	NSF	N-ethylmaleimide sensitive factor, vesicle fusing ATPase
927	2.23E-02	1.659	16843179	COPRS	coordinator of PRMT5 and differentiation stimulator
928	1.81E-02	1.658	16933667	NIPSNAP1	nipsnap homolog 1 (C. elegans)
929	3.40E-02	1.658	16878443	FOSL2	FOS like 2, AP-1 transcription factor subunit

930	4.98E-02	1.657	17112060	PHKA1	phosphorylase kinase regulatory subunit alpha 1
931	2.48E-02	1.657	16899263	MRPL53	mitochondrial ribosomal protein L53
932	4.44E-02	1.657	17112799	ARMCX2	armadillo repeat containing, X-linked 2
933	2.42E-02	1.656	17114007	APLN	apelin
934	1.03E-03	1.655	16834981	CRHR1	corticotropin releasing hormone receptor 1
935	1.32E-02	1.654	17011755	WISP3	WNT1 inducible signaling pathway protein 3
936	4.24E-02	1.653	16858970	ADGRE5	adhesion G protein-coupled receptor E5
937	2.67E-02	1.652	16875862	ZSCAN5A	zinc finger and SCAN domain containing 5A
938	2.94E-03	1.652	16782012	TRAJ58	T cell receptor alpha joining 58 (non-functional)
939	3.07E-02	1.652	16802918	PML	promyelocytic leukemia
940	4.74E-02	1.652	16723748	C11orf74	chromosome 11 open reading frame 74
941	3.83E-02	1.651	16734762	TRIM21	tripartite motif containing 21
942	2.55E-03	1.651	16874935	HAS1	hyaluronan synthase 1
943	4.36E-02	1.651	16674973	C1orf21	chromosome 1 open reading frame 21
944	3.04E-02	1.650	16957554	USF3	upstream transcription factor family member 3
945	4.82E-02	1.650	16857612	MCOLN1	mucolipin 1
946	2.54E-02	1.650	16703439	LINC00202-2	long intergenic non-protein coding RNA 202-2
947	2.35E-02	1.649	16845937	PLEKHM1	pleckstrin homology and RUN domain containing M1
948	2.30E-02	1.649	16826230	NETO2	neuropilin and tolloid like 2
949	4.89E-02	1.649	17048876	ARPC1A	actin related protein 2/3 complex subunit 1A
950	3.07E-02	1.648	16722603	LDHA	lactate dehydrogenase A
951	4.90E-02	1.647	17092712	PLIN2	perilipin 2
952	3.65E-02	1.647	16984244	OSMR	oncostatin M receptor
953	6.30E-03	1.646	16678752	DISC1	disrupted in schizophrenia 1
954	2.98E-02	1.645	16924007	DIP2A	disco interacting protein 2 homolog A
955	1.76E-02	1.643	16988423	PRR16	proline rich 16
956	8.63E-03	1.643	16804995	MAN2A2	mannosidase alpha class 2A member 2
957	3.68E-02	1.642	16773650	USPL1	ubiquitin specific peptidase like 1
958	1.88E-02	1.642	16810329	RPS27L	ribosomal protein S27 like
959	4.74E-02	1.642	16821585	GSE1	Gse1 coiled-coil protein
960	1.42E-02	1.641	16661567	PHACTR4	phosphatase and actin regulator 4
961	3.39E-02	1.641	16965041	DEFB131/LOC100129216	defensin beta 131
962	1.37E-02	1.640	17005276	SOX4	SRY-box 4
963	3.27E-02	1.639	16704844	TIMM23B	translocase of inner mitochondrial membrane 23 homolog B
964	2.14E-02	1.639	16998892	REEP5	receptor accessory protein 5
965	4.83E-02	1.639	16753030	PIP4K2C	phosphatidylinositol-5-phosphate 4-kinase type 2 gamma

966	2.60E-02	1.638	16715133	PPA1	pyrophosphatase (inorganic) 1
967	4.65E-02	1.638	16739552	NXF1	nuclear RNA export factor 1
968	3.95E-03	1.638	16823570	CDIP1	cell death-inducing p53 target 1
969	4.22E-02	1.637	16733597	NTM-IT	NTM intronic transcript
970	1.55E-02	1.637	17044201	KLHL7	kelch like family member 7
971	1.29E-02	1.637	16907353	ALS2	ALS2, alsin Rho guanine nucleotide exchange factor
972	4.35E-02	1.636	16715361	P4HA1	prolyl 4-hydroxylase subunit alpha 1
973	4.88E-02	1.636	17070144	LOC105375905	uncharacterized LOC105375905
974	1.34E-02	1.636	16662525	ADPRHL2	ADP-ribosylhydrolase like 2
975	3.35E-02	1.635	16848453	SLC39A11	solute carrier family 39 member 11
976	1.64E-02	1.635	16861724	FAM98C	family with sequence similarity 98 member C
977	1.53E-02	1.634	17087308	TDRD7	tudor domain containing 7
978	4.09E-02	1.634	16693272	S100A10	S100 calcium binding protein A10
979	3.64E-02	1.633	16720416	TSPAN4	tetraspanin 4
980	6.72E-03	1.633	16676086	PPFIA4	PTPRF interacting protein alpha 4
981	4.10E-02	1.632	17001763	TNIP1	TNFAIP3 interacting protein 1
982	4.40E-02	1.632	16789149	TNFAIP2	TNF alpha induced protein 2
983	1.53E-03	1.631	16742963	SYTL2	synaptotagmin like 2
984	2.20E-02	1.631	16791059	PSME2	proteasome activator subunit 2
985	4.93E-02	1.631	16873751	NAPA	NSF attachment protein alpha
986	2.12E-02	1.631	16837308	ARSG	arylsulfatase G
987	3.06E-02	1.631	16986450	AGGF1	angiogenic factor with G-patch and FHA domains 1
988	3.03E-02	1.629	16823928	LITAF	lipopolysaccharide induced TNF factor
989	3.57E-02	1.629	16893531	BOK	BOK, BCL2 family apoptosis regulator
990	2.97E-02	1.628	16708061	ZFYVE27	zinc finger FYVE-type containing 27
991	2.42E-02	1.628	16955975	SUCLG2	succinate-CoA ligase GDP-forming beta subunit
992	3.53E-02	1.628	16782373	LOC102724814	uncharacterized LOC102724814
993	4.96E-03	1.628	16698786	C1orf132	chromosome 1 open reading frame 132
994	4.28E-03	1.627	16957525	SPICE1	spindle and centriole associated protein 1
995	2.11E-02	1.627	16774808	PHF11	PHD finger protein 11
996	3.27E-02	1.627	16826586	IRX3	iroquois homeobox 3
997	1.49E-02	1.626	16707184	IFIT3	interferon induced protein with tetratricopeptide repeats 3
998	1.38E-02	1.626	16826212	C16orf87	chromosome 16 open reading frame 87
999	4.47E-02	1.625	16985094	ZSWIM6	zinc finger SWIM-type containing 6
1000	3.44E-02	1.625	17050578	CAV1	caveolin 1
1001	4.31E-02	1.624	16698425	RBBP5	RB binding protein 5, histone lysine methyltransferase complex subunit

1002	4.95E-02	1.623	16913206	PHF20	PHD finger protein 20
1003	2.69E-02	1.623	16997574	HOMER1	homer scaffolding protein 1
1004	2.28E-02	1.623	17092794	HACD4	3-hydroxyacyl-CoA dehydratase 4
1005	4.38E-02	1.622	16846976	VEZF1	vascular endothelial zinc finger 1
1006	2.83E-02	1.622	16843635	TBC1D3 (includes others)	TBC1 domain family member 3
1007	1.13E-02	1.622	16973832	LRPAP1	LDL receptor related protein associated protein 1
1008	2.94E-02	1.621	17005348	MRS2	MRS2, magnesium transporter
1009	4.59E-02	1.620	16964316	HTT	huntingtin
1010	4.75E-02	1.619	16948589	YEATS2	YEATS domain containing 2
1011	3.89E-02	1.619	16948021	ECT2	epithelial cell transforming 2
1012	4.34E-03	1.619	17108745	DHRSX	dehydrogenase/reductase X-linked
1013	3.77E-02	1.619	17096304	ANKRD18CP	ankyrin repeat domain 18C, pseudogene
1014	4.10E-02	1.618	17102111	PRDX4	peroxiredoxin 4
1015	1.23E-02	1.617	17101856	NHS	NHS actin remodeling regulator
1016	3.04E-02	1.617	16891723	MFF	mitochondrial fission factor
1017	3.97E-02	1.616	17022754	LAMA4	laminin subunit alpha 4
1018	2.29E-02	1.616	16708780	CNNM2	cyclin and CBS domain divalent metal cation transport mediator 2
1019	2.22E-02	1.616	17048538	CASD1	CAS1 domain containing 1
1020	2.50E-02	1.616	16795021	ANGEL1	angel homolog 1
1021	1.86E-02	1.614	17022150	POPDC3	popeye domain containing 3
1022	2.93E-02	1.614	17042936	NUDT1	nudix hydrolase 1
1023	1.38E-02	1.613	16699066	TMEM206	transmembrane protein 206
1024	4.02E-02	1.612	16887313	KLHL23/PHOSPHO2-KLHL23	kelch like family member 23
1025	3.28E-02	1.610	17009132	TMEM63B	transmembrane protein 63B
1026	4.33E-02	1.610	16765900	SMARCC2	SWI/SNF related, matrix associated,actin dependent reg of chromatin subfamily c member 2
1027	3.87E-02	1.610	16994890	DROSHA	drosha ribonuclease III
1028	4.74E-02	1.610	16718840	CACUL1	CDK2 associated cullin domain 1
1029	1.64E-02	1.609	16669708	LOC100996763/NOTCH2NL	notch 2 N-terminal like
1030	2.79E-02	1.609	16910020	HDAC4	histone deacetylase 4
1031	4.90E-02	1.608	16927907	BCR	BCR, RhoGEF and GTPase activating protein
1032	1.31E-02	1.607	16710017	ATE1-AS1	ATE1 antisense RNA 1
1033	1.41E-03	1.606	16903356	ZEB2	zinc finger E-box binding homeobox 2
1034	4.29E-02	1.605	16904586	TTC21B	tetratricopeptide repeat domain 21B

1035	4.62E-02	1.605	17092045	RFX3	regulatory factor X3
1036	4.05E-02	1.605	16667456	PTBP2	polypyrimidine tract binding protein 2
1037	1.44E-02	1.605	16702047	NET1	neuroepithelial cell transforming 1
1038	4.96E-02	1.604	16764620	SLC11A2	solute carrier family 11 member 2
1039	1.37E-02	1.604	17086418	LOC389765	kinesin family member 27 pseudogene
1040	3.48E-02	1.603	16912492	TM9SF4	transmembrane 9 superfamily member 4
1041	1.24E-02	1.603	16822356	LUC7L	LUC7 like
1042	3.17E-02	1.603	16747568	DSTNP2	destrin, actin depolymerizing factor pseudogene 2
1043	4.70E-02	1.603	16808974	COPS2	COP9 signalosome subunit 2
1044	4.18E-03	1.603	16938656	CMTM7	CKLF like MARVEL transmembrane domain containing 7
1045	3.54E-03	1.602	17006222	HLA-L	major histocompatibility complex, class I, L (pseudogene)
1046	4.36E-02	1.601	16774727	FNDC3A	fibronectin type III domain containing 3A
1047	4.72E-02	1.599	16847343	WFDC21P	WAP four-disulfide core domain 21, pseudogene
1048	3.32E-02	1.599	17019056	TREM1	triggering receptor expressed on myeloid cells 1
1049	4.93E-02	1.598	16687985	TM2D1	TM2 domain containing 1
1050	2.64E-02	1.596	16747529	TPI1	triosephosphate isomerase 1
1051	7.94E-03	1.594	17111413	FAM120C	family with sequence similarity 120C
1052	6.35E-03	1.593	17091444	TRAF2	TNF receptor associated factor 2
1053	3.47E-02	1.593	16796667	SLC25A29	solute carrier family 25 member 29
1054	3.91E-02	1.593	16789452	INF2	inverted formin, FH2 and WH2 domain containing
1055	4.32E-02	1.591	16665687	DNAJC6	DnaJ heat shock protein family (Hsp40) member C6
1056	8.52E-03	1.591	16904105	CD302	CD302 molecule
1057	3.02E-02	1.590	17022216	RTN4IP1	reticulon 4 interacting protein 1
1058	2.33E-02	1.589	16979317	C4orf3	chromosome 4 open reading frame 3
1059	1.51E-03	1.588	16815985	MKL2	MKL1/myocardin like 2
1060	1.33E-02	1.588	17007257	HLA-DRA	major histocompatibility complex, class II, DR alpha
1061	4.32E-02	1.588	16981266	DDX60L	DEAD-box helicase 60-like
1062	2.60E-02	1.587	16993127	TMED9	transmembrane p24 trafficking protein 9
1063	3.59E-02	1.587	16898601	NFU1	NFU1 iron-sulfur cluster scaffold
1064	3.93E-02	1.587	16779232	INTS6	integrator complex subunit 6
1065	8.52E-03	1.586	16981219	DDX60	DEXD/H-box helicase 60
1066	4.17E-02	1.585	16719838	TUBGCP2	tubulin gamma complex associated protein 2
1067	2.13E-02	1.585	17077502	TOX	thymocyte selection associated high mobility group box
1068	3.55E-02	1.585	16918455	PXMP4	peroxisomal membrane protein 4
1069	3.23E-02	1.585	16929562	HMOX1	heme oxygenase 1
1070	4.48E-02	1.584	17097369	ZNF883	zinc finger protein 883

1071	2.65E-02	1.583	17094671	LOC105376070	uncharacterized LOC105376070
1072	3.82E-02	1.583	16698789	C1orf132	chromosome 1 open reading frame 132
1073	5.20E-03	1.582	16793024	ERO1A	endoplasmic reticulum oxidoreductase 1 alpha
1074	7.64E-03	1.582	17110148	BCOR	BCL6 corepressor
1075	2.23E-02	1.581	16786060	LOC145474	uncharacterized LOC145474
1076	3.62E-02	1.581	16670383	HIST2H2AA3/HIST2H2AA4	histone cluster 2, H2aa3
1077	1.90E-02	1.580	17071776	OXR1	oxidation resistance 1
1078	4.14E-02	1.579	16980364	ZNF827	zinc finger protein 827
1079	4.53E-02	1.579	16992809	HIGD2A	HIG1 hypoxia inducible domain family member 2A
1080	2.47E-03	1.579	16657506	FAM87B	family with sequence similarity 87 member B
1081	4.34E-02	1.578	17074673	CTSB	cathepsin B
1082	3.01E-02	1.577	17082106	ZC3H3	zinc finger CCCH-type containing 3
1083	1.52E-02	1.577	16738544	UBE2L6	ubiquitin conjugating enzyme E2 L6
1084	7.21E-03	1.577	16758072	P2RX7	purinergic receptor P2X 7
1085	2.50E-02	1.577	16799357	EIF2AK4	eukaryotic translation initiation factor 2 alpha kinase 4
1086	2.23E-02	1.577	17105712	BEX3	brain expressed X-linked 3
1087	2.89E-02	1.576	16960442	PFN2	profilin 2
1088	3.33E-02	1.576	16981405	HPF1	histone PARylation factor 1
1089	4.42E-02	1.575	17049076	ZKSCAN1	zinc finger with KRAB and SCAN domains 1
1090	3.48E-02	1.575	16740854	RBM4B	RNA binding motif protein 4B
1091	4.16E-02	1.575	16922418	LINC00310	long intergenic non-protein coding RNA 310
1092	1.27E-02	1.574	17106640	ZBTB33	zinc finger and BTB domain containing 33
1093	1.22E-02	1.574	16986734	FAM151B	family with sequence similarity 151 member B
1094	4.38E-02	1.573	16724922	CTNND1	catenin delta 1
1095	3.14E-02	1.573	16857143	CCDC94	coiled-coil domain containing 94
1096	2.33E-02	1.572	17095525	CDK20	cyclin dependent kinase 20
1097	4.08E-02	1.571	17056372	LOC401320	uncharacterized LOC401320
1098	2.62E-02	1.571	16834921	HEXIM1	hexamethylene bisacetamide inducible 1
1099	2.94E-02	1.571	16991210	GM2A	GM2 ganglioside activator
1100	4.33E-02	1.571	16716239	GLUD1	glutamate dehydrogenase 1
1101	3.52E-02	1.571	16786367	COQ6	coenzyme Q6, monooxygenase
1102	4.31E-02	1.570	16939203	XYLB	xylulokinase
1103	4.11E-02	1.570	16935955	NUP50-AS1	NUP50 antisense RNA 1 (head to head)
1104	8.97E-03	1.570	16997662	DHFR	dihydrofolate reductase
1105	1.56E-02	1.570	16999432	C5orf63	chromosome 5 open reading frame 63
1106	2.80E-02	1.569	16878731	EHD3	EH domain containing 3

1107	1.48E-02	1.569	16719644	BNIP3	BCL2 interacting protein 3
1108	6.00E-03	1.568	16997646	LOC105379049	uncharacterized LOC105379049
1109	3.34E-02	1.567	16882277	VAMP8	vesicle associated membrane protein 8
1110	2.88E-02	1.567	16840703	FXR2	FMR1 autosomal homolog 2
1111	6.97E-03	1.567	16800450	B2M	beta-2-microglobulin
1112	2.61E-02	1.566	16994329	FAM173B	family with sequence similarity 173 member B
1113	2.12E-02	1.564	16881514	ALMS1	ALMS1, centrosome and basal body associated protein
1114	4.17E-03	1.563	16858546	ZNF439	zinc finger protein 439
1115	7.73E-03	1.563	17108113	PLXNB3	plexin B3
1116	4.48E-03	1.563	16990331	PCDHB15	protocadherin beta 15
1117	3.90E-02	1.563	16860709	GPI	glucose-6-phosphate isomerase
1118	1.85E-02	1.563	16662636	DNALI1	dynein axonemal light intermediate chain 1
1119	1.88E-02	1.562	16664852	CPT2	carnitine palmitoyltransferase 2
1120	4.56E-02	1.561	16974670	QDPR	quinoid dihydropteridine reductase
1121	4.26E-02	1.561	16808318	PPIP5K1	diphosphoinositol pentakisphosphate kinase 1
1122	3.62E-02	1.560	16739836	TRPT1	tRNA phosphotransferase 1
1123	4.84E-02	1.560	17076609	SFRP1	secreted frizzled related protein 1
1124	4.78E-02	1.560	16744078	NPAT	nuclear protein, coactivator of histone transcription
1125	4.79E-02	1.560	16827805	COG4	component of oligomeric golgi complex 4
1126	2.56E-02	1.558	16766465	NDUFA4L2	NDUFA4, mitochondrial complex associated like 2
1127	1.80E-02	1.558	16704107	LINC00839	long intergenic non-protein coding RNA 839
1128	7.47E-03	1.558	16692341	ACP6	acid phosphatase 6, lysophosphatidic
1129	1.98E-03	1.557	16662945	MFSD2A	major facilitator superfamily domain containing 2A
1130	4.32E-02	1.556	16790364	RAB2B	RAB2B, member RAS oncogene family
1131	2.22E-02	1.556	17065630	mir-597	microRNA 597
1132	4.73E-02	1.554	16879817	TTC7A	tetratricopeptide repeat domain 7A
1133	2.27E-02	1.554	17096402	TRMO	tRNA methyltransferase O
1134	3.90E-02	1.554	17084493	GALT	galactose-1-phosphate uridylyltransferase
1135	4.62E-02	1.553	16671178	SNAPIN	SNAP associated protein
1136	4.48E-02	1.552	16798136	SNRPN	small nuclear ribonucleoprotein polypeptide N
1137	3.91E-02	1.552	17056160	HOXA9	homeobox A9
1138	2.83E-02	1.551	17088462	PAPPA	pappalysin 1
1139	3.77E-02	1.551	17047176	GTF2I	general transcription factor IIi
1140	3.74E-02	1.549	16847586	STRADA	STE20-related kinase adaptor alpha
1141	4.46E-02	1.549	17018329	MLN	motilin
1142	4.98E-02	1.548	16849788	SLC38A10	solute carrier family 38 member 10

1143	1.00E-02	1.547	16909413	SP110	SP110 nuclear body protein
1144	4.95E-02	1.547	16987610	RGMB	repulsive guidance molecule family member b
1145	8.61E-03	1.547	16936925	LRRN1	leucine rich repeat neuronal 1
1146	1.65E-02	1.546	16720231	PHRF1	PHD and ring finger domains 1
1147	4.04E-02	1.546	17009071	MAD2L1BP	MAD2L1 binding protein
1148	6.94E-03	1.545	16829894	PLD2	phospholipase D2
1149	2.90E-02	1.545	16829242	GALNS	galactosamine (N-acetyl)-6-sulfatase
1150	1.85E-02	1.544	17113710	TMEM255A	transmembrane protein 255A
1151	4.45E-02	1.544	16844061	PLXDC1	plexin domain containing 1
1152	4.09E-02	1.544	16835045	LRRC37A3 (includes others)	leucine rich repeat containing 37 member A3
1153	2.03E-02	1.544	17101896	CDKL5	cyclin dependent kinase like 5
1154	4.63E-02	1.543	16987046	TMEM161B-AS1	TMEM161B antisense RNA 1
1155	3.15E-02	1.543	17014421	LOC729603	calcineurin like EF-hand protein 1 pseudogene
1156	6.23E-03	1.543	16849585	CANT1	calcium activated nucleotidase 1
1157	2.96E-02	1.542	17048228	FZD1	frizzled class receptor 1
1158	4.32E-02	1.541	16851026	IMPA2	inositol monophosphatase 2
1159	3.91E-02	1.541	17052306	ADCK2	aarF domain containing kinase 2
1160	4.08E-02	1.540	17085507	LOC102725139	uncharacterized LOC102725139
1161	3.04E-02	1.539	16865175	NDUFA3	NADH:ubiquinone oxidoreductase subunit A3
1162	1.94E-02	1.539	16907281	ALS2CR11	amyotrophic lateral sclerosis 2 chromosome region candidate 11
1163	4.81E-02	1.538	17079380	MTERF3	mitochondrial transcription termination factor 3
1164	6.25E-03	1.538	16705754	ADAMTS14	ADAM metallopeptidase with thrombospondin type 1 motif 14
1165	3.47E-02	1.537	16886417	LYPD6B	LY6/PLAUR domain containing 6B
1166	3.02E-02	1.537	16918976	DSN1	DSN1 homolog, MIS12 kinetochore complex component
1167	3.87E-02	1.536	17043702	TMEM106B	transmembrane protein 106B
1168	4.22E-02	1.536	16720331	LOC171391	uncharacterized LOC171391
1169	4.30E-02	1.535	17005248	E2F3	E2F transcription factor 3
1170	7.52E-04	1.534	16738933	VWCE	von Willebrand factor C and EGF domains
1171	3.99E-02	1.534	16921289	HELZ2	helicase with zinc finger 2
1172	3.16E-02	1.534	16727462	BBS1	Bardet-Biedl syndrome 1
1173	5.47E-04	1.533	16766923	PPM1H	protein phosphatase, Mg2+/Mn2+ dependent 1H
1174	2.29E-02	1.533	17117884	CYP4F62P	cytochrome P450 family 4 subfamily F member 62, pseudogene
1175	1.73E-02	1.532	16938092	SH3BP5-AS1	SH3BP5 antisense RNA 1
1176	3.04E-02	1.532	16745654	OR8B12	olfactory receptor family 8 subfamily B member 12
1177	4.12E-02	1.532	16788534	mir-127	microRNA 127

1178	9.92E-03	1.532	16729240	EMSY	EMSY, BRCA2 interacting transcriptional repressor
1179	2.72E-02	1.532	16938112	BTD	biotinidase
1180	3.63E-02	1.531	16992897	TSPAN17	tetraspanin 17
1181	3.26E-02	1.531	16697843	TMEM9	transmembrane protein 9
1182	3.73E-02	1.531	16776117	PCCA	propionyl-CoA carboxylase alpha subunit
1183	3.52E-02	1.531	16805287	MIR3175	microRNA 3175
1184	4.47E-02	1.530	17066897	SLC25A37	solute carrier family 25 member 37
1185	4.70E-02	1.530	16730061	CEP295	centrosomal protein 295
1186	2.95E-02	1.527	16843658	TBC1D3 (includes others)	TBC1 domain family member 3
1187	4.03E-02	1.527	16723326	QSER1	glutamine and serine rich 1
1188	4.96E-02	1.527	16723353	DEPDC7	DEP domain containing 7
1189	3.70E-02	1.526	16876609	TRAPPC12	trafficking protein particle complex 12
1190	4.92E-02	1.525	16865675	RPL28	ribosomal protein L28
1191	2.48E-02	1.525	16752586	NABP2	nucleic acid binding protein 2
1192	7.61E-03	1.524	16968680	SPP1	secreted phosphoprotein 1
1193	3.49E-02	1.524	17073721	MROH1	maestro heat like repeat family member 1
1194	2.94E-02	1.524	16921258	EEF1A2	eukaryotic translation elongation factor 1 alpha 2
1195	2.47E-02	1.523	16889503	STRADB	STE20-related kinase adaptor beta
1196	4.83E-03	1.523	16793585	C14orf39	chromosome 14 open reading frame 39
1197	2.74E-02	1.522	17074097	LOC101927752	uncharacterized LOC101927752
1198	2.12E-02	1.522	17075922	KIF13B	kinesin family member 13B
1199	3.60E-02	1.521	16790776	HOMEZ	homeobox and leucine zipper encoding
1200	4.11E-02	1.521	16920762	CTSZ	cathepsin Z
1201	2.78E-02	1.520	17093441	FAM219A	family with sequence similarity 219 member A
1202	2.85E-02	1.519	16957041	LINC00882	long intergenic non-protein coding RNA 882
1203	3.13E-02	1.519	17101129	ASMTL-AS1	ASMTL antisense RNA 1
1204	1.47E-02	1.519	17051827	AKR1B10	aldo-keto reductase family 1 member B10
1205	4.71E-02	1.519	16707143	ACTA2-AS1	ACTA2 antisense RNA 1
1206	2.23E-02	1.518	16884335	BCL2L11	BCL2 like 11
1207	4.02E-03	1.517	16795128	TMED8	transmembrane p24 trafficking protein family member 8
1208	2.29E-02	1.516	16851900	MAPRE2	microtubule associated protein RP/EB family member 2
1209	1.81E-02	1.515	17046998	ELN	elastin
1210	4.58E-02	1.515	17003320	DOK3	docking protein 3
1211	3.24E-02	1.514	16993302	ZFP2	ZFP2 zinc finger protein
1212	2.05E-02	1.514	17117609	LOC101927016	keratin-associated protein 21-1-like
1213	3.87E-02	1.514	16780509	DOCK9	dedicator of cytokinesis 9

1214	3.39E-02	1.514	16915015	CSTF1	cleavage stimulation factor subunit 1
1215	2.07E-02	1.513	16838879	MRPL12	mitochondrial ribosomal protein L12
1216	3.87E-02	1.512	17097308	PTBP3	polypyrimidine tract binding protein 3
1217	2.13E-02	1.512	16864059	CD37	CD37 molecule
1218	3.79E-02	1.512	16965190	CC2D2A	coiled-coil and C2 domain containing 2A
1219	3.34E-02	1.511	17000532	SIL1	SIL1 nucleotide exchange factor
1220	3.92E-02	1.511	16864331	MED25	mediator complex subunit 25
1221	2.42E-02	1.511	17117913	BRWD1-IT2	BRWD1 intronic transcript 2 (non-protein coding)
1222	4.20E-02	1.510	16662338	ZMYM1	zinc finger MYM-type containing 1
1223	4.57E-02	1.510	16927731	IGLV1-50	immunoglobulin lambda variable 1-50 (non-functional)
1224	3.71E-02	1.510	16890675	IGFBP2	insulin like growth factor binding protein 2
1225	3.95E-02	1.510	17057066	COA1	cytochrome c oxidase assembly factor 1 homolog
1226	4.65E-02	1.509	16660527	ZBTB40	zinc finger and BTB domain containing 40
1227	4.02E-02	1.509	16699112	NSL1	NSL1, MIS12 kinetochore complex component
1228	1.19E-02	1.509	17090938	LOC105376311	uncharacterized LOC105376311
1229	2.55E-02	1.509	16827420	GFOD2	glucose-fructose oxidoreductase domain containing 2
1230	3.27E-02	1.508	17012245	NKAIN2	Na+/K+ transporting ATPase interacting 2
1231	3.60E-03	1.508	16900998	LYG1	lysozyme g1
1232	4.69E-02	1.508	16802854	CD276	CD276 molecule
1233	4.53E-02	1.507	16749382	MED21	mediator complex subunit 21
1234	4.51E-02	1.507	16670309	FAM231D/LOC100996721	family with sequence similarity 231 member D
1235	4.63E-03	1.506	16798240	SNRPN	small nuclear ribonucleoprotein polypeptide N
1236	3.76E-02	1.506	16911238	CRLS1	cardiolipin synthase 1
1237	2.43E-02	1.505	17020990	LOC101928570	uncharacterized LOC101928570
1238	1.05E-02	1.505	16971465	FAM160A1	family with sequence similarity 160 member A1
1239	1.61E-02	1.505	16951917	CMTM6	CKLF like MARVEL transmembrane domain containing 6
1240	2.15E-02	1.505	16852445	C18orf54	chromosome 18 open reading frame 54
1241	3.12E-02	1.504	16901894	ZC3H8	zinc finger CCCH-type containing 8
1242	4.41E-02	1.504	17003953	SCGB3A1	secretoglobin family 3A member 1
1243	4.94E-02	1.503	16995989	FGF10	fibroblast growth factor 10
1244	1.54E-02	1.502	16759872	LOC105369597	uncharacterized LOC105369597
1245	4.02E-02	1.501	17098071	NDUFA8	NADH:ubiquinone oxidoreductase subunit A8
1246	4.66E-02	-1.503	17075238	LOC105379316	uncharacterized LOC105379316
1247	4.64E-02	-1.504	16894155	LOC105373405	uncharacterized LOC105373405
1248	3.26E-02	-1.507	16975045	LOC105374556	uncharacterized LOC105374556
1249	2.17E-02	-1.509	17006745	APOM	apolipoprotein M

1250	4.62E-03	-1.515	16980245	GYPE	glycophorin E (MNS blood group)
1251	4.22E-03	-1.524	16877290	MIR3681	microRNA 3681
1252	4.99E-02	-1.524	16891609	LOC105373907	uncharacterized LOC105373907
1253	2.53E-02	-1.527	16896335	MYADML	myeloid associated differentiation marker like (pseudogene)
1254	3.90E-03	-1.527	16900180	IGKV3D-7	immunoglobulin kappa variable 3D-7
1255	3.75E-04	-1.531	17008463	NCR2	natural cytotoxicity triggering receptor 2
1256	2.03E-03	-1.538	16910536	PDCD1	programmed cell death 1
1257	3.33E-02	-1.544	16827741	SMG1P7	SMG1P7, nonsense mediated mRNA decay associated PI3K related kinase pseudogene 7
1258	3.22E-02	-1.557	16927861	IGLJ4	immunoglobulin lambda joining 4 (non-functional)
1259	4.96E-02	-1.557	16990483	ARHGAP26	Rho GTPase activating protein 26
1260	5.49E-03	-1.558	16944784	MYLK-AS1	MYLK antisense RNA 1
1261	2.70E-03	-1.559	16846254	HOXB5	homeobox B5
1262	3.24E-02	-1.561	16900140	IGK	immunoglobulin kappa locus
1263	4.24E-02	-1.562	16865112	MYADM	myeloid associated differentiation marker
1264	4.62E-02	-1.565	16666055	CTH	cystathionine gamma-lyase
1265	3.80E-02	-1.571	17109923	FTHL17	ferritin heavy chain like 17
1266	2.92E-02	-1.577	16696580	LOC102724601	uncharacterized LOC102724601
1267	2.72E-02	-1.584	16902199	EN1	engrailed homeobox 1
1268	1.83E-02	-1.585	16795567	KCNK10	potassium two pore domain channel subfamily K member 10
1269	3.45E-02	-1.586	16965798	PCDH7	protocadherin 7
1270	2.23E-02	-1.590	16917797	LOC105372562	uncharacterized LOC105372562
1271	1.61E-03	-1.591	16729986	FAT3	FAT atypical cadherin 3
1272	2.92E-03	-1.591	16888786	C2orf88	chromosome 2 open reading frame 88
1273	3.87E-03	-1.594	16958890	LOC653712	intraflagellar transport 122 homolog (Chlamydomonas) pseudogene
1274	2.77E-02	-1.594	17071773	LOC105375699	uncharacterized LOC105375699
1275	1.95E-03	-1.594	16938989	ITGA9	integrin subunit alpha 9
1276	7.83E-03	-1.596	16683903	NR0B2	nuclear receptor subfamily 0 group B member 2
1277	2.99E-02	-1.596	16784787	DAAM1	dishevelled associated activator of morphogenesis 1
1278	3.55E-03	-1.598	16783602	SSTR1	somatostatin receptor 1
1279	3.49E-02	-1.607	17015581	LOC101928106	uncharacterized LOC101928106
1280	3.66E-02	-1.611	16839519	TLCD2	TLC domain containing 2
1281	4.30E-02	-1.618	16922851	SH3BGR	SH3 domain binding glutamate rich protein
1282	1.05E-02	-1.623	16928411	IGLL3P	immunoglobulin lambda like polypeptide 3, pseudogene
1283	1.87E-02	-1.623	16851309	GREB1L	growth regulation by estrogen in breast cancer 1 like
1284	3.02E-03	-1.626	16751554	KRT18	keratin 18
1285	3.49E-02	-1.627	17060944	MYL10	myosin light chain 10

1286	1.08E-02	-1.635	16864393	ATF5	activating transcription factor 5
1287	1.12E-03	-1.638	16973498	SPON2	spondin 2
1288	4.37E-02	-1.641	17098066	TTLL11-IT1	
1289	4.34E-02	-1.641	16788659	SNORD114-12	small nucleolar RNA, C/D box 114-12
1290	4.89E-02	-1.641	16946597	LOC105374138	uncharacterized LOC105374138
1291	1.55E-02	-1.643	16880277	CCDC85A	coiled-coil domain containing 85A
1292	1.09E-03	-1.645	16866394	PLPP2	phospholipid phosphatase 2
1293	3.27E-02	-1.647	16799739	CHAC1	ChaC glutathione specific gamma-glutamylcyclotransferase 1
1294	2.84E-02	-1.649	16790360	SNORD9	small nucleolar RNA, C/D box 9
1295	3.39E-02	-1.660	16718779	EMX2OS	EMX2 opposite strand/antisense RNA
1296	1.84E-02	-1.661	16657758	LOC105378585	uncharacterized LOC105378585
1297	3.94E-02	-1.663	16819176	MIR3935	microRNA 3935
1298	4.14E-02	-1.668	16844600	KRTAP4-11	keratin associated protein 4-11
1299	2.66E-02	-1.668	16946077	IL20RB	interleukin 20 receptor subunit beta
1300	2.19E-02	-1.671	17061298	RELN	reelin
1301	4.54E-02	-1.672	16773086	FGF9	fibroblast growth factor 9
1302	2.53E-02	-1.675	17092331	PTPRD	protein tyrosine phosphatase, receptor type D
1303	4.96E-02	-1.678	17044069	mir-1183	microRNA 1183
1304	4.86E-02	-1.679	16839683	OR3A2	olfactory receptor family 3 subfamily A member 2
1305	3.75E-02	-1.684	17096805	LOC340512	uncharacterized LOC340512
1306	1.67E-02	-1.684	16751381	ACVRL1	activin A receptor like type 1
1307	5.75E-03	-1.687	16927172	mir-185	microRNA 185
1308	1.94E-02	-1.687	16759810	LOC102723544	uncharacterized LOC102723544
1309	5.36E-03	-1.688	17020089	GSTA3	glutathione S-transferase alpha 3
1310	1.55E-02	-1.694	16963113	APOD	apolipoprotein D
1311	4.86E-03	-1.697	16711023	TUBB8	tubulin beta 8 class VIII
1312	2.89E-02	-1.700	16807139	MEIS2	Meis homeobox 2
1313	3.25E-02	-1.705	17014015	mir-1202	microRNA 1202
1314	3.23E-02	-1.705	17050765	KCND2	potassium voltage-gated channel subfamily D member 2
1315	3.51E-02	-1.708	16718244	LOC101927523	uncharacterized LOC101927523
1316	1.36E-03	-1.711	16994081	IRX2	iroquois homeobox 2
1317	1.39E-02	-1.712	16950440	OXTR	oxytocin receptor
1318	1.13E-02	-1.730	17069577	C8orf46	chromosome 8 open reading frame 46
1319	4.54E-02	-1.732	16952975	PRSS50	protease, serine 50
1320	1.22E-02	-1.744	16852871	SERPINB2	serpin family B member 2
1321	4.14E-03	-1.744	17100201	CLIC3	chloride intracellular channel 3

1322	3.18E-02	-1.753	16865036	mir-515	microRNA 520c
1323	3.04E-03	-1.754	16846218	HOXB2	homeobox B2
1324	1.73E-02	-1.756	16865050	mir-515	microRNA 520c
1325	9.71E-03	-1.763	17080188	SYBU	syntabulin
1326	4.93E-02	-1.765	16725533	PGA5 (includes others)	pepsinogen 5, group I (pepsinogen A)
1327	8.37E-03	-1.779	16714084	MIR4294	microRNA 4294
1328	2.86E-02	-1.781	17017625	TNXA	tenascin XA (pseudogene)
1329	2.16E-02	-1.790	17010825	LINC01590	long intergenic non-protein coding RNA 1590
1330	5.37E-04	-1.792	16897872	LOC100129434	uncharacterized LOC100129434
1331	4.98E-02	-1.807	17002612	SLIT3	slit guidance ligand 3
1332	5.29E-03	-1.807	16927821	IGLV2-11	immunoglobulin lambda variable 2-11
1333	3.73E-02	-1.811	17012447	LAMA2	laminin subunit alpha 2
1334	1.47E-02	-1.824	16718077	SH3PXD2A	SH3 and PX domains 2A
1335	1.06E-02	-1.839	16910609	TRIB3	tribbles pseudokinase 3
1336	2.11E-02	-1.848	17084025	LINC01239	long intergenic non-protein coding RNA 1239
1337	2.06E-02	-1.851	17096855	ACTL7B	actin like 7B
1338	1.88E-04	-1.854	16709739	EMX2	empty spiracles homeobox 2
1339	3.81E-02	-1.870	17002194	ADAM19	ADAM metallopeptidase domain 19
1340	8.92E-03	-1.882	16835436	LOC105371809	uncharacterized LOC105371809
1341	1.11E-02	-1.885	16669454	EMBP1	embigin pseudogene 1
1342	1.23E-02	-1.889	16865062	mir-515	microRNA 520c
1343	1.96E-02	-1.894	16795965	FBLN5	fibulin 5
1344	3.07E-02	-1.897	16954343	RBM5-AS1	RBM5 antisense RNA 1
1345	2.89E-02	-1.928	17106438	DOCK11	dedicator of cytokinesis 11
1346	4.41E-04	-1.931	16763577	SLC38A4	solute carrier family 38 member 4
1347	6.13E-03	-1.951	16887484	ERICH2	glutamate rich 2
1348	3.07E-02	-1.956	16997383	F2RL2	coagulation factor II thrombin receptor like 2
1349	1.08E-02	-1.960	16797419	IGHJ1	immunoglobulin heavy joining 1
1350	5.70E-03	-1.969	16814738	TMEM204	transmembrane protein 204
1351	2.82E-03	-1.974	16947683	LOC105374191	uncharacterized LOC105374191
1352	7.46E-04	-1.993	16852858	SERPINB7	serpin family B member 7
1353	3.51E-02	-1.998	16709936	PLPP4	phospholipid phosphatase 4
1354	2.01E-02	-2.000	16724995	OR1S1	olfactory receptor family 1 subfamily S member 1 (gene/pseudogene)
1355	4.06E-02	-2.005	16759218	ADGRD1	adhesion G protein-coupled receptor D1
1356	3.26E-02	-2.024	16732043	MIR4492	microRNA 4492
1357	3.20E-02	-2.029	16697196	FAM129A	family with sequence similarity 129 member A

1358	1.56E-02	-2.035	16788528	mir-665	microRNA 665
1359	1.06E-02	-2.035	16894283	MBOAT2	membrane bound O-acyltransferase domain containing 2
1360	3.05E-02	-2.065	16779667	PCDH9	protocadherin 9
1361	2.20E-02	-2.072	16844564	KRTAP1-4	keratin associated protein 1-4
1362	1.03E-03	-2.073	17016369	HIST1H2BB	histone cluster 1, H2bb
1363	1.67E-02	-2.077	16865082	mir-515	microRNA 520c
1364	4.97E-02	-2.080	16872926	PSG5	pregnancy specific beta-1-glycoprotein 5
1365	2.36E-02	-2.098	16854437	CDH2	cadherin 2
1366	1.51E-02	-2.125	17056281	CHN2	chimerin 2
1367	3.08E-02	-2.129	17094475	LOC105379818	uncharacterized LOC105379818
1368	4.40E-03	-2.161	16737344	PAMR1	peptidase domain containing associated with muscle regeneration 1
1369	8.83E-03	-2.161	16687799	DAB1	DAB1, reelin adaptor protein
1370	3.95E-04	-2.168	17059355	SEMA3D	semaphorin 3D
1371	1.45E-04	-2.189	16669105	MAB21L3	mab-21 like 3
1372	3.61E-04	-2.192	16846259	HOXB6	homeobox B6
1373	2.25E-03	-2.198	17095887	ASPN	asporin
1374	6.55E-03	-2.199	16991988	CTB-78F1.2	uncharacterized LOC101927885
1375	3.67E-03	-2.206	16798665	GOLGA8J (includes others)	golgin A8 family member R
1376	1.58E-03	-2.207	16841340	MYH2	myosin heavy chain 2
1377	6.41E-03	-2.208	16850595	EMILIN2	elastin microfibril interfacer 2
1378	2.10E-04	-2.221	17062127	WNT2	Wnt family member 2
1379	9.18E-04	-2.241	16972663	TENM3	teneurin transmembrane protein 3
1380	2.31E-03	-2.262	16904484	SNORA70F	small nucleolar RNA, H/ACA box 70F
1381	6.98E-03	-2.273	17002657	mir-218	microRNA 218-2
1382	4.97E-02	-2.284	16870453	COMP	cartilage oligomeric matrix protein
1383	2.18E-02	-2.296	16798404	SNORD115-30	small nucleolar RNA, C/D box 115-30
1384	5.75E-04	-2.298	16846226	HOXB3	homeobox B3
1385	8.52E-05	-2.309	16873562	PTGIR	prostaglandin I2 (prostacyclin) receptor (IP)
1386	1.18E-02	-2.327	16769569	NUAK1	NUAK family kinase 1
1387	5.06E-03	-2.332	16775763	mir-622	microRNA 622
1388	1.61E-02	-2.344	16848173	ABCA9	ATP binding cassette subfamily A member 9
1389	3.58E-02	-2.358	17103748	mir-500	microRNA 501
1390	3.55E-02	-2.361	17054530	MIR4655	microRNA 4655
1391	1.61E-04	-2.391	17002667	FAM196B	family with sequence similarity 196 member B
1392	3.03E-02	-2.394	16842266	MFAP4	microfibrillar associated protein 4
1393	9.88E-05	-2.410	16713729	SYT15	synaptotagmin 15

1394	8.94E-03	-2.413	16777455	C1QTNF9B-AS1	C1QTNF9B antisense RNA 1
1395	9.57E-05	-2.414	16956316	CNTN3	contactin 3
1396	2.91E-04	-2.427	17059388	KIAA1324L	KIAA1324 like
1397	1.87E-03	-2.449	16896859	LOC101929723	uncharacterized LOC101929723
1398	2.99E-03	-2.492	17101990	CNKSR2	connector enhancer of kinase suppressor of Ras 2
1399	3.84E-04	-2.511	16935380	CHADL	chondroadherin like
1400	3.80E-02	-2.522	16986409	F2R	coagulation factor II thrombin receptor
1401	2.11E-03	-2.535	16999475	FBN2	fibrillin 2
1402	2.23E-02	-2.601	16997802	HAPLN1	hyaluronan and proteoglycan link protein 1
1403	5.75E-04	-2.608	17044692	PRR15	proline rich 15
1404	1.52E-04	-2.636	17106200	MIR3978	microRNA 3978
1405	1.75E-02	-2.650	16848219	ABCA6	ATP binding cassette subfamily A member 6
1406	3.94E-03	-2.742	16888270	ITGA4	integrin subunit alpha 4
1407	8.65E-04	-2.801	16968122	FRAS1	Fraser extracellular matrix complex subunit 1
1408	3.31E-03	-2.817	16937855	FBLN2	fibulin 2
1409	6.31E-03	-2.852	16964799	SORCS2	sortilin related VPS10 domain containing receptor 2
1410	3.76E-04	-2.852	17114334	mir-503	microRNA 503
1411	1.37E-02	-3.113	16788628	SNORD114-6	small nucleolar RNA, C/D box 114-6
1412	1.07E-04	-3.175	17114326	MIR503HG	MIR503 host gene
1413	1.04E-02	-3.285	16964831	mir-4798	microRNA 4798
1414	6.30E-03	-3.306	17070013	RDH10	retinol dehydrogenase 10 (all-trans)
1415	2.05E-02	-3.474	17088821	OR1L6	olfactory receptor family 1 subfamily L member 6
1416	2.36E-03	-3.681	17047795	CD36	CD36 molecule
1417	2.36E-03	-3.756	16697471	B3GALT2	beta-1,3-galactosyltransferase 2
1418	3.99E-02	-3.789	16904667	SCN9A	sodium voltage-gated channel alpha subunit 9
1419	2.09E-03	-4.148	17087413	GALNT12	polypeptide N-acetylgalactosaminyltransferase 12
1420	4.11E-02	-4.413	16882744	IGKV1D-27	immunoglobulin kappa variable 1D-27 (pseudogene)
1421	6.30E-04	-4.709	17087430	COL15A1	collagen type XV alpha 1 chain
1422	1.46E-04	-5.246	16773131	SGCG	sarcoglycan gamma
1423	1.31E-05	-5.720	16734355	H19	H19, imprinted maternally expressed transcript (non-protein coding)
1424	2.42E-03	-6.479	16980762	SFRP2	secreted frizzled related protein 2
1425	2.71E-04	-6.758	17007982	PI16	peptidase inhibitor 16
